# Identification of NS2 determinants stimulating intrinsic HCV NS2 protease activity

**DOI:** 10.1371/journal.ppat.1010644

**Published:** 2022-06-21

**Authors:** Olaf Isken, Thomas Walther, Luis Wong-Dilworth, Dirk Rehders, Lars Redecke, Norbert Tautz

**Affiliations:** 1 Institute of Virology and Cell Biology, University of Luebeck, Luebeck, Germany; 2 Institute of Biochemistry, University of Luebeck, Luebeck, Germany; 3 Deutsches Elektronen Synchrotron (DESY), Photon Science, Hamburg, Germany; Duke University Medical Center, UNITED STATES

## Abstract

Hepatitis C Virus NS2-NS3 cleavage is mediated by NS2 autoprotease (NS2^pro^) and this cleavage is important for genome replication and virus assembly. Efficient NS2-NS3 cleavage relies on the stimulation of an intrinsic NS2^pro^ activity by the NS3 protease domain. NS2^pro^ activation depends on conserved hydrophobic NS3 surface residues and yet unknown NS2-NS3 surface interactions. Guided by an *in silico* NS2-NS3 precursor model, we experimentally identified two NS2 surface residues, F103 and L144, that are important for NS2^pro^ activation by NS3. When analyzed in the absence of NS3, a combination of defined amino acid exchanges, namely F103A and L144I, acts together to increase intrinsic NS2^pro^ activity. This effect is conserved between different HCV genotypes. For mutation L144I its stimulatory effect on NS2^pro^ could be also demonstrated for two other mammalian hepaciviruses, highlighting the functional significance of this finding. We hypothesize that the two exchanges stimulating the intrinsic NS2^pro^ activity mimic structural changes occurring during NS3-mediated NS2^pro^ activation. Introducing these activating NS2^pro^ mutations into a NS2-NS5B replicon reduced NS2-NS3 cleavage and RNA replication, indicating their interference with NS2-NS3 surface interactions pivotal for NS2^pro^ activation by NS3. Data from chimeric hepaciviral NS2-NS3 precursor constructs, suggest that NS2 F103 is involved in the reception or transfer of the NS3 stimulus by NS3 P115. Accordingly, fine-tuned NS2-NS3 surface interactions are a salient feature of HCV NS2-NS3 cleavage. Together, these novel insights provide an exciting basis to dissect molecular mechanisms of NS2^pro^ activation by NS3.

## Author summary

The replication strategy of many viruses involves the translation of a large polyprotein that is processed by viral and/or cellular proteases. This genome organization has many benefits for the virus; it allows the condensation of genetic information, as well as temporal and spatial regulation of protein activity depending on polyprotein cleavage events. Because viral precursor proteins have important functions during all aspects of viral life cycle, including genome replication and virion morphogenesis, a better understanding of polyprotein cleavage regulation and the structure/function of their precursor proteins is necessary. However, many determinants of such regulation are still unknown. Here we define such determinants for NS2^pro^ activation by its cofactor NS3, required for efficient NS2-NS3 cleavage of the HCV polyprotein. This protease activation within the HCV polyprotein involves specific NS2:NS3 surface contacts that most likely result in an optimized geometry of the NS2^pro^ active site. These results offer novel insights into the modes of HCV polyprotein processing.

## Introduction

Worldwide approximately 70 million people are chronically infected by hepatitis C virus (HCV) and are at risk of developing severe liver disease including fibrosis, cirrhosis, and hepatocellular carcinoma. While the treatment options of HCV infections have been improved due to the new era of direct acting antiviral agents (DAAs), HCV-related mortality and morbidity is expected to increase due to ageing of the infected population [1]. There is no vaccine available, so major challenges in basic, translational, and clinical research remain.

HCV belongs to the *Hepacivirus* genus of the *Flaviviridae* family and is still the sole member of the hepaciviruses that infect humans. However, multiple other hepaciviruses were discovered recently from diverse host ranges, including horses, cattle, rodents, bats, and New and Old-World primates [2]. The HCV genome consists of a 9.6 kb positive-strand RNA molecule and encodes a polyprotein that is co- and post-translationally cleaved by cellular and viral proteases. Host signal peptidase is processing the N-terminally encoded Core, E1, E2, and p7 [3,4]. NS2-NS3 autoprocessing is mediated by the NS2 cysteine protease [5,6]. The nonstructural polyprotein NS3-NS5B is processed by NS3 serine protease and its cofactor, NS4A [7].

HCV NS2 is a transmembrane protein consisting of an N-terminal domain with three transmembrane helices and a C-terminal domain encoding a cysteine protease [8–10]. According to structural studies and reporter assays, NS2 forms a dimeric protease with two composite active sites in which one monomer contributes histidine and glutamate residues and the other contributes the cysteine residue [8,11]. NS2 proteins from nonhuman hepaciviruses are also dimeric cysteine proteases that form composite active sites and exhibit intrinsic proteolytic activity, similar to HCV NS2 [11]. The activity of the NS2 cysteine protease catalytic domain is regulated by the NS3 N-terminal domain [8–13]. Recently, a conserved hydrophobic surface area in the NS3 N-terminal protease domain was identified that activates NS2 protease to mediate efficient NS2-NS3 cleavage [13]. Studies with related hepaciviruses demonstrated the importance of similarly located hydrophobic surface residues for the NS3-mediated NS2 protease activation in nonhuman hepaciviruses [11,14]. Interestingly, the NS2 protease was shown to exhibit efficient intrinsic proteolytic activity in the absence of NS3 moiety when placed in the context of C-terminal tag fusions via flexible linkers [11]. These findings indicate that the NS3 protease domain acts as a more complex regulatory cofactor for hepaciviral NS2 protease to control the critical NS2-NS3 cleavage. Together, these observations suggest hydrophobic NS2-NS3 surface interactions are a commonly shared unique mode of NS2 protease regulation in hepaciviruses.

Whereas NS2 is dispensable for HCV RNA replication [15], inhibiting NS2-NS3 autoprocessing by either mutating the NS2 catalytic residues, inhibiting NS2 palmitoylation or by blocking NS3-mediated NS2 activation was shown to impair HCV replication by reducing the availability of free NS3 [13,16,17]. Accordingly, this unique mode of NS2 protease regulation shows the functional importance of this cleavage event in the hepacivirus life cycle. Interestingly, the NS2-activating NS3 surface area is also promoting alternative protein-protein interactions required for NS5A hyperphosporylation, replicase assembly, and genome replication [13]. Despite its function during polyprotein processing, HCV NS2 also plays a critical role in HCV particle assembly. It has been shown that NS2 promotes HCV assembly by recruiting the viral envelope proteins to the virus assembly sites via interactions with both structural (E1 and E2) and nonstructural (NS3 and NS5A) proteins [10,18–21].

Although much progress has been made in our understanding of NS2-NS3 biology, key questions concerning intramolecular NS2:NS3 interactions and structural changes connected to NS2^pro^ activation, remain poorly understood. This is mainly due to the lack of structural information of the uncleaved NS2-NS3 precursor protein [8,22]. Especially, the NS2 determinants that interact with NS3 during NS2^pro^ activation have not been characterized so far.

In this work, we identified NS2 surface residues in close proximity to the NS2 protease active site as determinants of the NS3-mediated NS2^pro^ activation. Mechanistic characterization of this process led to the identification of two NS2 surface residues (F103 and L144), which, when mutated, were able to increase the intrinsic NS2^pro^ activity in the absence of NS3. Their functional importance was emphasized by the demonstration that their stimulating character is conserved among different HCV genotypes. A F103 and L144 permutation analysis revealed striking differences between these determinants suggesting that both residues contribute differently to an increased intrinsic NS2^pro^ activity.

Interestingly, these activating NS2 mutations, when introduced into a NS2-NS5B replicon, were reducing NS2-NS3 cleavage and RNA replication efficiency. This observation indicates that these mutations, while stimulating NS2 protease in the absence of NS3, might interfere with critical NS2-NS3 surface interactions during efficient NS2-NS3 cleavage. Accordingly, we could identify two pivotal NS2-determinants for the intrinsic NS2 protease stimulation that, in the absence of NS3, mimic structural changes occurring during NS3-mediated NS2 protease stimulation. Therefore, this study provides an exciting basis to dissect molecular mechanism(s) of NS2 activation by NS3 in more detail.

## Results

The HCV NS2-NS3 site is cleaved by the NS2 cysteine auto-protease [8]. To do so efficiently, the intrinsic proteolytic activity of NS2 is stimulated by the NS3 protease domain functioning as its activating cofactor [8–13]. Recently, it was demonstrated that this activation depends on conserved hydrophobic surface residues (Y105, P115 and L127) of NS3 protease domain and that activation is a shared mode of NS2 protease regulation among different mammalian hepaciviruses [11,13]. However, the NS2 determinants important for NS2^pro^ activation by NS3 are still unknown.

### Identification of NS2 surface determinants for NS3-mediated NS2 protease activation by alanine mutagenesis based on a hypothetical NS2-NS3 model

**Fig 1:**
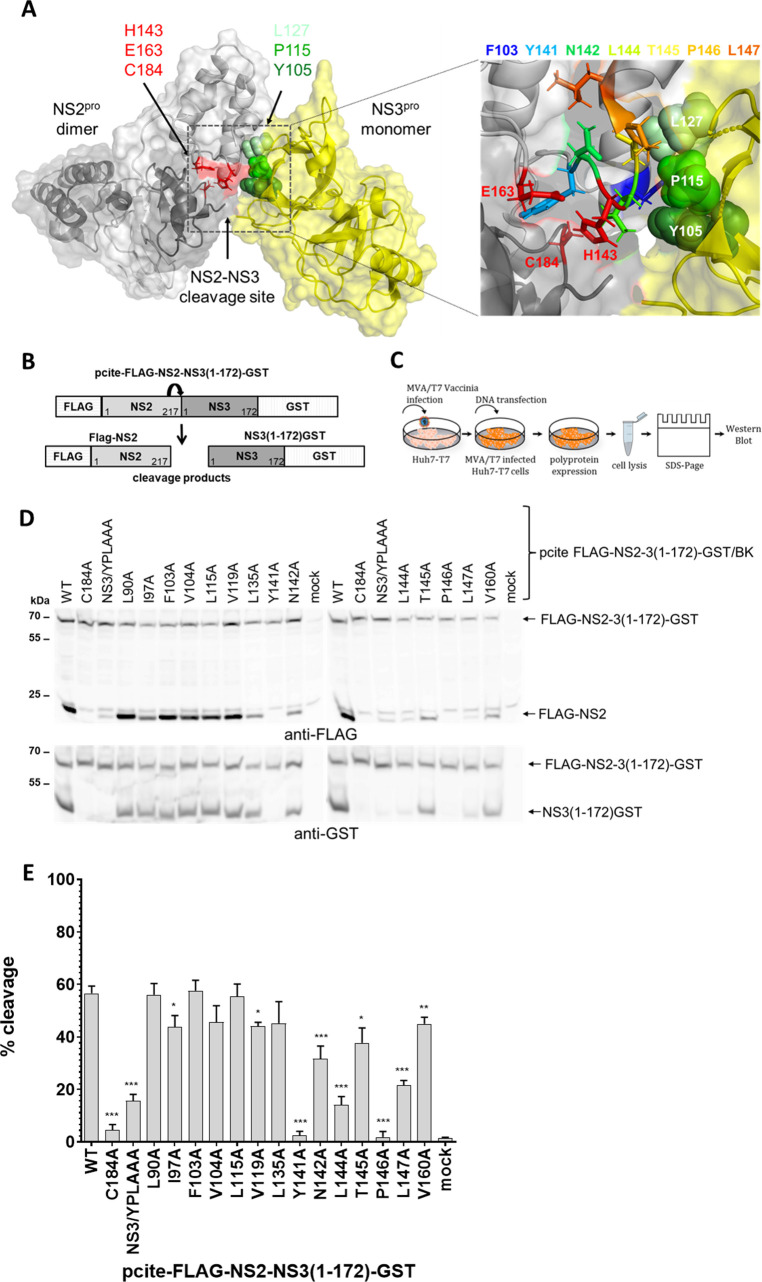
Mapping of NS2 determinants important for NS3-mediated NS2^pro^ activation by alanine scanning mutagenesis. (**A**) Hypothetical model of NS2^pro^-NS3^pro^ prior to NS2-NS3 cleavage. Structural model of a hypothetical NS2^pro^-NS3^pro^ precursor protein consisting of two NS2^pro^ (light and dark gray, respectively) and one NS3^pro^ molecules (yellow). The overall structure is shown in surface representation. Carbon and backbone ribbon are colored in light and dark grey for the two NS2^pro^ domains and yellow for the NS3^pro^ domain, respectively. Post-cleavage structures of NS2^pro^ (residues 94–217, pdb id 2HD0, [8]) and the crystal structure of the NS3 protease domain complexed with a synthetic NS4A cofactor peptide (NS3 residues 1–180, pdb id 1A1R, [24]) representing protein sequences from Hepatitis C virus genotype 1a (isolate H77) have been used to generate this model. One NS2^pro^ composite active site is shown with H143, E163 and C184 in red stick representation. The NS2^pro^-activating NS3 surface residues Y105, P115 and L127 are shown in different green spheres, respectively. The position of the NS2-NS3 cleavage site is indicated. An enlargement of the proposed interacting surface area between NS2 residues in proximity to the NS2^pro^ active site (indicated in red stick representation) and the activating NS3^pro^ surface residues consisting of Y105, P115 and L127 is shown on the right. NS2 residues close to the NS2^pro^ active site that were analyzed for their ability to functionally interact with activating NS3^pro^ surface residues during NS2^pro^ stimulation by NS3^pro^ are shown by stick representation and their identities are given by rainbow coloring. NS2 ^pro^ residues I97, V104, L115, V119, L135 and V160, which were mutated as controls are not indicated in the model for clarity of the figure. (**B**) Scheme of NS2-NS3 cleavage analysis using FLAG-NS2-NS3(1–172)-GST expression (left). (**C**) Analysis of NS2-mediated cleavage of FLAG-NS2-NS3(1–172)-GST polyprotein fragments by the MVA/T7^pol^ expression system in Huh7-T7 cells. (**D**) Western blot analysis of NS2-NS3 cleavage after expression of FLAG-NS2-NS3(1–172)-GST polyprotein fragments. One representative blot from three independent experiments is shown. Positions of the uncleaved precursor protein FLAG-NS2-NS3(1–172)-GST and cleavage products FLAG-NS2 and NS3(1–172)-GST are indicated by arrows on the right. (**E**) NS2^pro^ cleavage efficiencies. Signals of uncleaved FLAG-NS2-NS3(1–172)-GST and the cleavage products FLAG-NS2 and NS3(1–172)-GST were quantified by ImageJ software from Western blots to calculate the percentage of NS2-NS3 cleavage. Mean and standard deviations of NS2^pro^ cleavage efficiencies from three independent experiments are presented. WT: wild-type; mock: transfection control without plasmid DNA. Molecular mass standards are shown on the left. Asterisks indicate statistically significant differences in comparison to WT (* p < 0.05; ** p < 0.01; *** p < 0.001).

We proposed that hydrophobic NS2-NS3 surface interactions are salient features for NS2 activation by NS3 [13]. Crucial for a better understanding of the process is the identification of the NS2 determinants required for NS2^pro^ activation. While structural information of the NS2^pro^ domain and the NS3 protein are available such information for the uncleaved NS2-NS3 precursor is still missing [8,23]. To overcome this lack of information, we generated an *in silico* NS2-NS3 model based on post cleavage structures of the NS2^pro^ and NS3^pro^ domains representing protein sequences from hepatitis C virus genotype 1a (isolate H77), respectively (Fig 1A). We hypothesized that the stimulating hydrophobic NS3 surface residues Y105, P115 and L127 interact with NS2 surface residues in the proximity of the NS2^pro^ active site to allow NS2^pro^ activation by NS3. In addition, we placed in our NS2-NS3 precursor protein model also the NS2-NS3 cleavage site in proximity to the NS2^pro^ active site (see Materials and Methods section for details).

The results of the energetic minimization of this NS2-NS3 precursor model revealed a potential NS2^pro^:NS3^pro^ interaction region: surface residues in proximity to NS2^pro^ active site H143 could potentially form interactions with the activating NS3^pro^ surface residues Y105, P115 and L127 (Fig 1A). It is noteworthy, that F103, which is very exposed, would be able to form potential interactions with any of the three activating NS3^pro^ surface residues. Furthermore, NS2 L144 could potentially interact with NS3 Y105 or P115, respectively. In this NS2-NS3 precursor model, L144 directly faces P115 of NS3, forming hydrophobic contacts ranging between 5 and 7 Å, while F103 could form stacking interactions (π-π-interactions) with Y105 in NS3, at a distance of approximately 4 Å for the aromatic ring structures. In addition, hydrophobic interactions of F103 with the side chains of P115 and L127 are also possible at a distance of approximately 4–5 Å. According to this NS2-NS3 precursor model, the buried surface for the NS2-NS3 interface was calculated to be approx. 1050 Å^2^ (Fig 1A). Interestingly, the strict conservation of NS2 F103 and L144 among different HCV genotypes points to their potential functional importance.

To test the importance of these hydrophobic NS2 residues for NS3-mediated NS2^pro^ stimulation, we mutated residues F103 and L144 to alanine. The mutagenesis was performed using construct pcite-FLAG-NS2-NS3(1–172)-GST/BK (HCV genotype 1b) previously used to study HCV NS2-NS3 cleavage (Fig 1B, [13]). In addition, we mutated several other residues that are either in close proximity to the NS2^pro^ active site (Y141, N142, T145, P146 and L147) or are more distantly located to the active site residues (L90, I97, V104, L115, V119, L135 and V160). Among the selected residues targeted by mutagenesis (Fig 1) most of them are either conserved across different HCV genotypes (F103, L144, Y141, P146, V160) or have a conserved hydrophobic character (V104, L115, V119, L135, L147).

The NS2/C184A mutant with an inactive NS2 protease served as negative control. We also included the NS3/YPL-AAA mutant (NS3/Y105A-P115A-L127A) that is unable to stimulate NS2^pro^ to show the extent of NS2-NS3 cleavage without NS3 activation [13]. Accordingly, WT-like NS2-NS3 cleavage efficiencies would mark residues not critical for NS2 protease activation, while mutations with an impact on NS2-NS3 cleavage comparable to the NS3/YPL-AAA mutant (NS3/Y105A-P115A-L127A) would identify NS2 amino acids potentially involved in the NS3-mediated NS2^pro^ activation process. We analyzed NS2-NS3 cleavage in a replication-independent cell-based cleavage assay based on Huh7-T7 cells and T7 RNA polymerase-driven expression of FLAG-NS2-NS3(1–172)GST (Fig 1C and [13]). The uncleaved FLAG-NS2-NS3(1–172)-GST/BK polyprotein fragment and the products of NS2-mediated cleavage were separated by SDS-PAGE and analyzed by Western blot using anti-FLAG and anti-GST antibodies, respectively (Fig 1D). Western blot signal intensities for uncleaved FLAG-NS2-NS3(1–172)-GST and cleaved FLAG-NS2 were determined to calculate the individual NS2-NS3 cleavage rates by quantification of signal intensities using the Odyssey SA imaging system (LI-COR) (Fig 1E). As expected, WT FLAG-NS2-NS3(1–172)-GST/BK exhibits a strong NS2-NS3 cleavage, while C184A mutant did not show detectable cleavage. In the case of NS3/YPL-AAA we only detected residual NS2^pro^ activity marking the intrinsic NS2^pro^ activity in the absence of NS3 activation [13]. The alanine scanning mutagenesis revealed that most of the tested NS2 mutations had no (L90A, I97A, F103A, V104A, L115A, V119A and V160A) or moderate negative (L135A, N142A, T145A) effects on NS2-NS3 cleavage (Fig 1D and 1E). In contrast, Y141A and P146A inhibited NS2-NS3 cleavage similar to our active site mutant NS2/C184A, indicating that either the active site configuration or NS2 protein conformation is affected. Interestingly, mutations L144A and L147A did show a low level of NS2-NS3 cleavage comparable to the one of mutant NS3/YPL-AAA known to block NS3-mediated NS2^pro^ stimulation (Fig 1D and 1E; [13]).

Taken together, this mutagenesis suggests that the L144 and L147 residues, that are in close proximity to the NS2^pro^ active site residue H143, may play a critical for the NS2^pro^ activation by NS3. The identification of L144 and L147 as potential NS2 determinants engaged in functional NS2:NS3 interactions during NS2^pro^ activation by NS3 suggests that our NS2-NS3 model represents a valuable tool to dissect mechanistic aspects of this protease activation process.

### Analysis of NS2 alanine surface mutations for NS2 cleavage in the absence of the NS3 cofactor revealed F103A mutation is stimulating the intrinsic NS2 autoprotease activity

**Fig 2:**
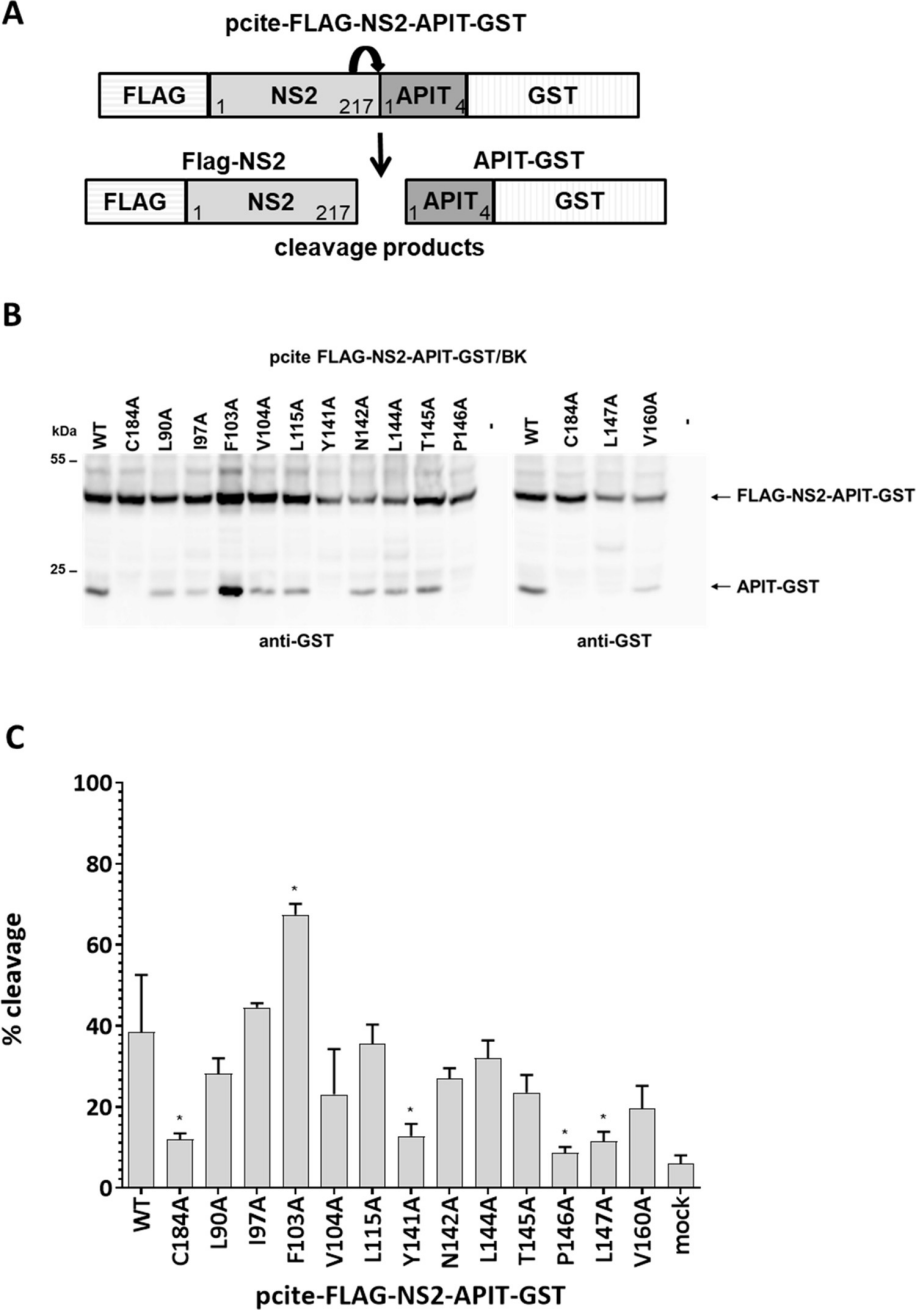
Re-analyzing of NS2^pro^ surface residues in the NS2-APIT-GST context revealed F103A as a stimulating determinant for the intrinsic NS2^pro^ activity in the absence of the NS3 cofactor. **(A)** Schematic representation of the pcite-FLAG-NS2-APIT-GST expression construct used to analyze the NS2^pro^ activity in the absence of its cofactor NS3. Cleavage of FLAG-NS2-APIT-GST results in generation of FLAG-NS2 and APIT-GST. **(B)** Indicated pcite-FLAG-NS2-APIT-GST plasmids were transfected into Huh7-T7 cells and expressed by the MVA/T7^pol^ expression system. One representative blot from three independent experiments is shown. Molecular mass standards are shown on the left. Positions of the FLAG-NS2-APIT-GST and the cleavage product APIT-GST are indicated on the right. WT: wild-type; mock: transfection control without DNA. (**C**) NS2 protease cleavage efficiencies. Signals of uncleaved FLAG-NS2-APIT-GST and the cleavage product APIT-GST were quantified by ImageJ software from three Western blots to calculate the percentage of FLAG-NS2-APIT-GST cleavage. Mean and standard deviations of NS2 cleavage efficiencies from three independent experiments are presented. Asterisk indicate statistically significant differences in comparison to WT (* p < 0.05).

The observed interference of the L144A or L147A mutations with the NS3-mediated NS2 stimulation during NS2-NS3 cleavage is indicating that these mutations might block crucial interactions between activating NS2 and NS3 surface residues critical for this process. However, the inefficient cleavage between NS2-NS3 could also originate from a diminished NS2^pro^ activity caused by a disruption of the NS2^pro^ active site configuration. Accordingly, we analyzed the set of NS2 mutations for their impact on the intrinsic NS2^pro^ activity in the absence of the NS3-cofactor to rule out direct effects on the NS2^pro^ not related to NS2^pro^ activation by NS3. For this purpose, we employed the construct pcite-FLAG-NS2-APIT-GST in which FLAG-NS2 is C-terminally fused to the N-terminal four amino acids of NS3 (APIT) to maintain the authentic NS2-NS3 cleavage site upstream of GST used for monitoring NS2^pro^ activity (Fig 2A).

Thus, cleavage of FLAG-NS2-APIT-GST is mediated by the intrinsic NS2^pro^ activity. All NS2^pro^ mutations were introduced into this plasmid, generating the respective pcite-FLAG-NS2-APIT-GST derivatives. Again, we used MVA/T7^pol^-infected Huh7-T7 cells to express wild-type and mutant FLAG-NS2-APIT-GST proteins to monitor NS2^pro^ cleavage efficiencies by Western blotting using anti-GST antibodies (Fig 2B).

As expected, wild-type FLAG-NS2-APIT-GST was cleaved to a lower percentage compared to FLAG-NS2-NS3(1–172)-GST/BK, which is characteristic for the lower intrinsic NS2^pro^ activity (Fig 2B, lane 1). No intrinsic activity was detected for the FLAG-NS2-APIT-GST/C184A negative control mutant, confirming the specificity of this assay. The NS2 mutations L90A, I97A, V104A, L115A, N124A, L144A T145A and V160A showed slightly reduced intrinsic NS2^pro^ activities in the absence of NS3 compared to WT (Fig 2B and 2C). Furthermore, the Y141A, P146A and L147A mutations either abolished (Y141A and P146A) or strongly reduced (L147A), intrinsic NS2^pro^ activity in the FLAG-NS2-APIT-GST context suggesting a detrimental effect on the NS2 protease. The observation that the L144A exchange allows for intrinsic NS2^pro^ cleavage activity comparable to wild-type is indicating that (i) the NS2^pro^ with this mutation is functional and (ii) its strongly reduced activity in the FLAG-NS2-NS3-GST context suggest its involvement in NS2^pro^ activation by NS3. Together these results suggest that L144 represents one NS2 surface residue important for the NS3-mediated NS2^pro^ activation.

However, another highly interesting observation from this analysis was that the mutation F103A led to an increased NS2^pro^ cleavage activity in the absence of the NS3 cofactor when compared to wild-type FLAG-NS2-APIT-GST (Fig 2B and 2C). An increased intrinsic NS2^pro^ activity could indicate that F103A exchange in FLAG-NS2-APIT-GST either enhances a better positioning of the cleavage site relative to the NS2^pro^ active site or allows for an optimized NS2^pro^ active site conformation in the absence of the stimulating NS3 cofactor.

### The NS2-L144 permutation analysis of the NS2 protease in the absence of NS3 cofactor revealed that L144I is stimulating the intrinsic NS2 protease activity

**Fig 3:**
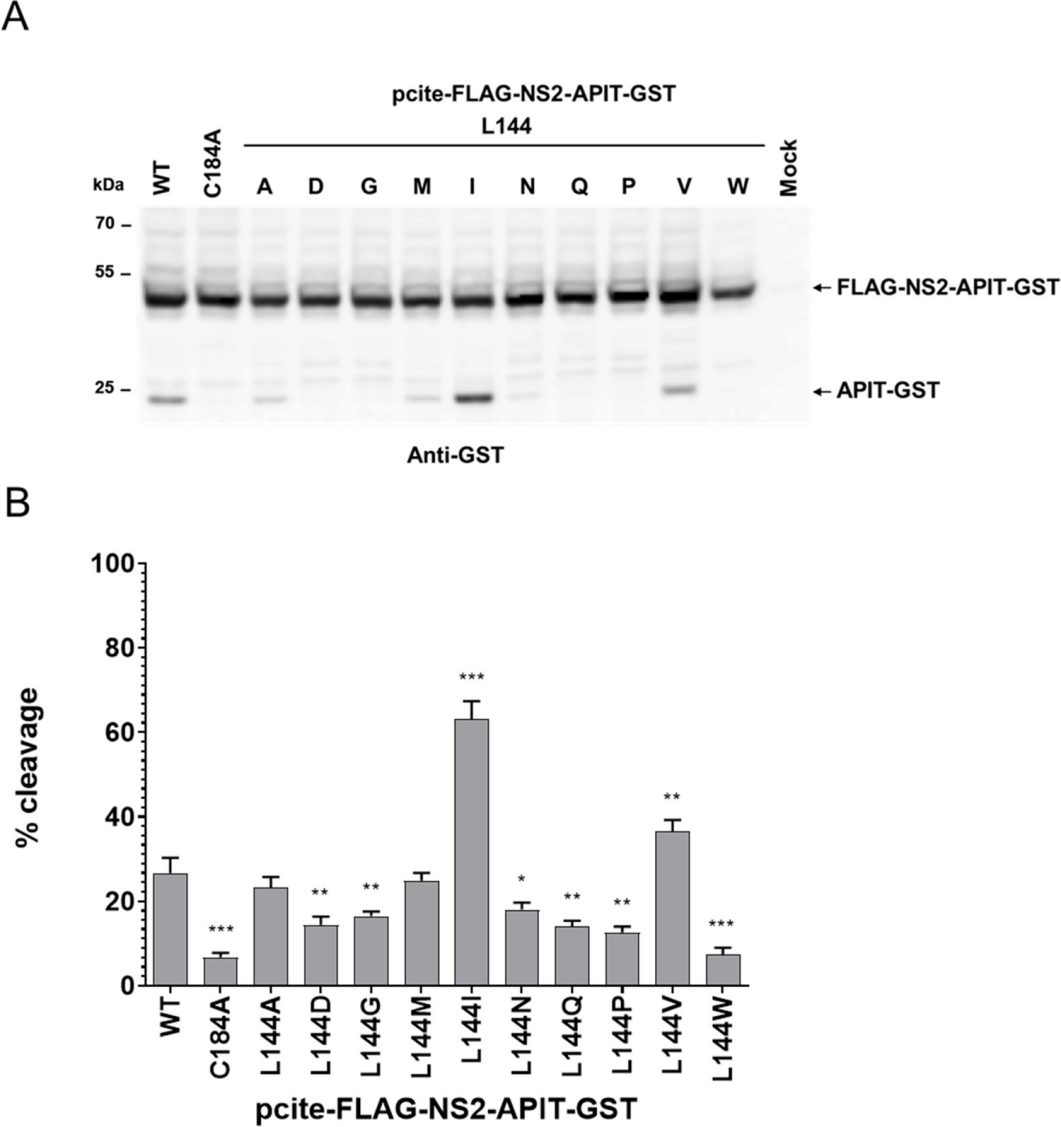
The amino acid exchange L144I is stimulating the intrinsic NS2^pro^ cleavage efficiency in the absence of the NS3 cofactor. **(A)** Analysis of the intrinsic NS2^pro^ activity assay in the MVA/T7^pol^ expression system in Huh7-T7 cells. NS2-mediated cleavage of FLAG-NS2-APIT-GST/BK was analyzed by Western blotting using GST-specific antibodies. One representative blot from three independent experiments is shown. **(B)** NS2^pro^ cleavage efficiencies. Signals of uncleaved FLAG-NS2-APIT-GST and the cleavage product APIT-GST were quantified by ImageJ software from three Western blots to calculate the percentage of NS2-APIT-GST cleavage. Mean and standard deviations of NS2^pro^ cleavage efficiencies from three independent experiments are presented. Molecular mass standards are shown on the left. Positions of the uncleaved precursor FLAG-NS2-APIT-GST and the cleavage product APIT-GST are indicated on the right. WT: wild-type; mock: transfection control without plasmid DNA. Asterisks indicate statistically significant differences in comparison to WT (* p < 0.05; ** p < 0.01; *** p < 0.001).

Our surprising finding that the F103A mutation increases the NS2^pro^ activity in the absence of the NS3^pro^ domain suggests that certain NS2 mutations have the ability to either induce optimizations of the cleavage site positioning and/or active site conformation in the absence of NS3 cofactor. Mechanistically, such mutations might mimic NS2 alterations induced by NS3 in the NS2-NS3 precursor resulting in efficient NS2^pro^ activity. Considering the importance of the L144 residue for NS2 activation by NS3 (Fig 1C), we next wanted to investigate whether the replacement of L144 by other amino acids also increase intrinsic NS2^pro^ activity. Accordingly, we analyzed a panel of NS2 L144 permutations in the context of pcite-FLAG-NS2-APIT-GST/BK for their impact on the intrinsic NS2^pro^ activity (Fig 3). The pcite-FLAG-NS2-APIT-GST/BK permutation derivatives were transfected into Huh7-T7 cells and NS2^pro^ activity was analyzed by Western blotting. The expression of FLAG-NS2-APIT-GST/WT and FLAG-NS2-APIT-GST/C184A served as positive and negative control for intrinsic NS2^pro^ activity, respectively.

While most of the analyzed L144 permutations (L144A, L144D, L144G, L144M and L144N, L144Q, L144P and L144W) interfered with the intrinsic NS2^pro^ activity in the FLAG-NS2-APIT-GST context to different degrees (Fig 3), exchanging L144 to isoleucine (L144I) is stimulating the intrinsic NS2^pro^ activity compared to WT (Fig 3). Accordingly, we identified two amino acid exchanges in the NS2^pro^ domain (F103A and L144I) that can stimulate the intrinsic NS2^pro^ activity.

### A comparison of NS2 F103 and NS2 L144 mutations revealed different requirements for intrinsic NS2 protease activity at these positions

Our finding that two different NS2 mutations can stimulate the intrinsic NS2 protease activity is intriguing since it could provide insight into the NS2 protease stimulation process. To also determine the amino acid characteristics critical for efficient intrinsic NS2 protease activity, we introduced F103 permutations into pcite-FLAG-NS2-APIT-GST to perform a limited permutation analysis for this amino acid. The pcite-FLAG-NS2-APIT-GST F103 permutation plasmids were transfected into Huh7-T7 cells and NS2 protease activity was analyzed by Western blotting with FLAG-NS2-APIT-GST/WT and FLAG-NS2-APIT-GST/C184A serving as positive and negative control, respectively.

**Fig 4:**
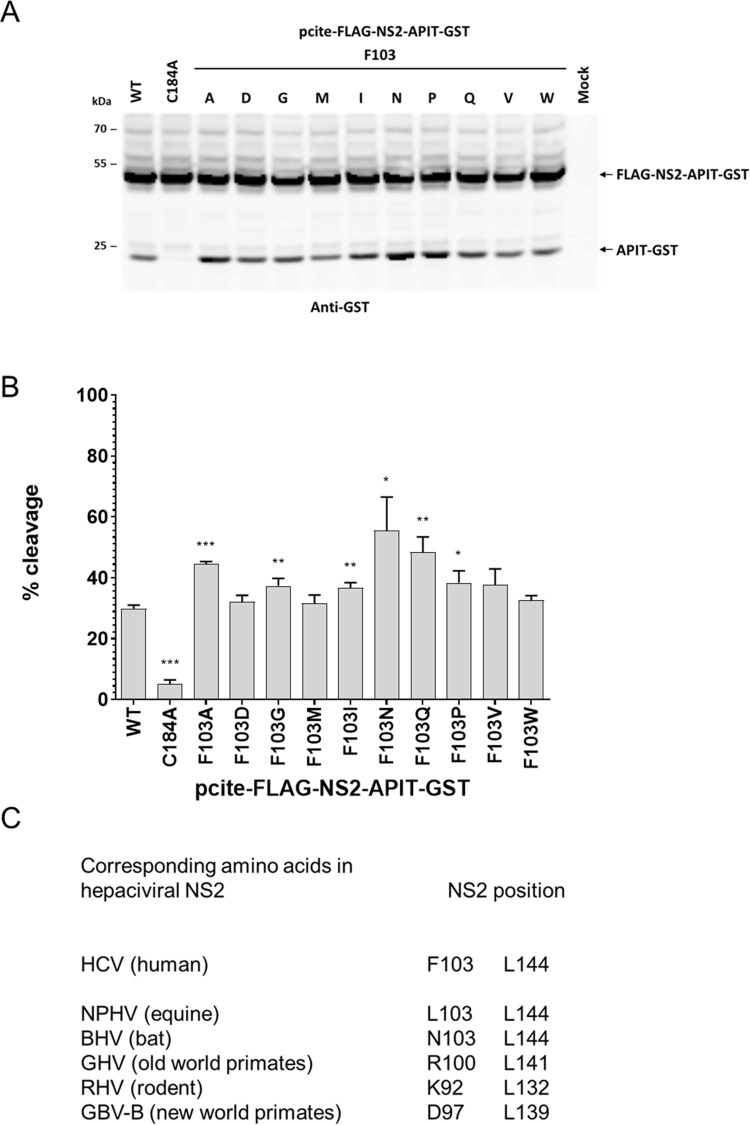
F103 permutation analysis revealed a broad spectrum of amino acids is allowing intrinsic NS2 protease cleavage. **(A)** Analysis of the intrinsic NS2^pro^ activity in the MVA/T7^pol^ expression system in Huh7-T7 cells. NS2-mediated cleavage of FLAG-NS2-APIT-GST was analyzed by Western blotting using GST-specific antibodies. **(B)** NS2^pro^ cleavage efficiencies. Signals of uncleaved FLAG-NS2-APIT-GST and the cleavage product APIT-GST were quantified by ImageJ from three Western blots to calculate the percentage of NS2-APIT-GST cleavage. Mean and standard deviations of NS2^pro^ cleavage efficiencies from three independent experiments are presented. Molecular mass standards are shown on the left. Positions of the uncleaved precursors FLAG-NS2-APIT-GST and the cleavage product APIT-GST are indicated on the right. WT: wild-type; mock: transfection control without plasmid DNA. **(C)** Conservation of HCV F103- and L144-corresponding NS2 residues in indicated mammalian hepacivirus species representatives are shown [11]. Asterisks indicate statistically significant differences in comparison to WT (* p < 0.05; ** p < 0.01; *** p < 0.001).

In contrast to the strict requirement for branched aliphatic amino acids observed for NS2 position 144, all analyzed F103 permutations showed a similar or slightly higher (F103A, F103N, F103Q, F103P) intrinsic NS2 protease activity when compared to WT (Fig 4). Accordingly, a broad functional flexibility for NS2 amino acid 103 exists to allow intrinsic NS2 protease activity. Thus, these results highlight striking differences regarding the required amino acid characteristics at these two positions for NS2 protease functionality.

These observations are in line with conservation of these position in NS2 sequences of different mammalian hepaciviruses (NS2_hepaci_): Leucine is highly conserved at positions corresponding to NS2_HCV_ L144 while at the position corresponding to NS2_HCV_ F103- a wide range of amino acids can be found in different NS2_hepaci_ sequences (Fig 4C; [11]). It is tempting to speculate that these divergent amino acid requirements suggest NS2 F103 and L144 serve different tasks to support the intrinsic NS2^pro^ activity and or its stimulation by NS3.

### The combination of F103A and L144I exchanges is increasing the stimulation of the intrinsic NS2 protease activity in different HCV genotypes

**Fig 5:**
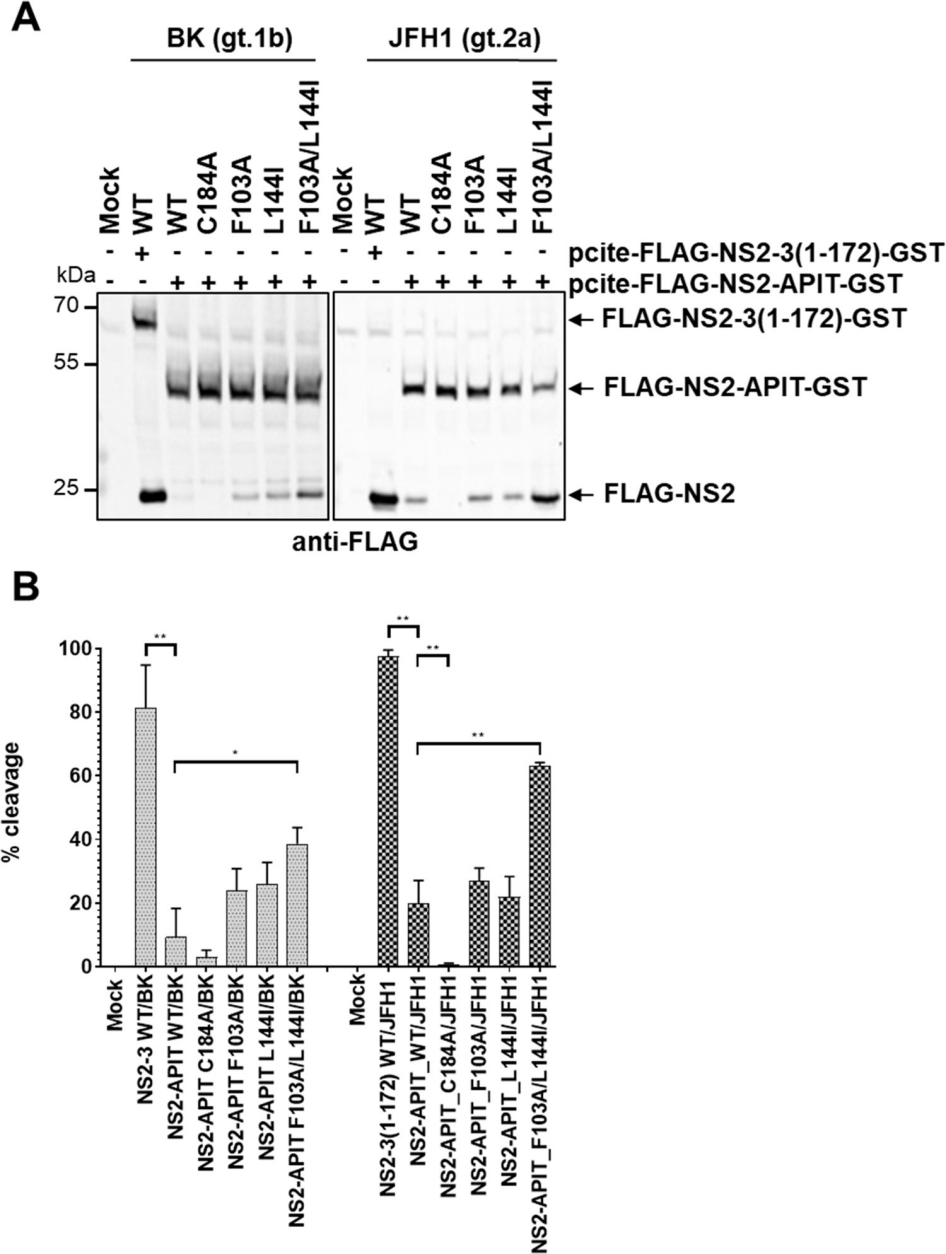
The amino acid exchanges F103A and L144I lead to a stimulation of the intrinsic NS2^pro^ cleavage efficiency in the absence of the NS3 cofactor in two different HCV genotypes. **(A)** Analysis of the intrinsic NS2^pro^ activity in the MVA/T7^pol^ expression system in Huh7-T7 cells. NS2-mediated cleavage of FLAG-NS2-NS3(1–172)-GST or FLAG-NS2-APIT-GST polyprotein fragments was analyzed by Western blotting using FLAG-specific antibodies. One representative blot from three independent experiments is shown. Molecular mass standards are shown on the left. Positions of the uncleaved precursors FLAG-NS2-NS3(1–172)-GST or FLAG-NS2-APIT-GST and cleavage product FLAG-NS2 are indicated by arrows on the right. **(B)** Quantification of NS2-protease mediated cleavage efficiencies (percent of cleavage calculated as percent of cleavage product over the sum of uncleaved precursor plus cleaved product) from lysates subjected to immunoblotting using infrared fluorescent-labeled secondary antibodies and the Odyssey SA imaging system (*LI-COR*). Background was subtracted from FLAG-specific signals of uncleaved precursors and cleavage product using the corresponding signal of the mock sample. Mean and standard deviation of NS2^pro^ cleavage efficiencies from three independent experiments are presented. WT: wild-type; mock: transfection control without plasmid DNA. Asterisks indicate statistically significant differences in comparison to WT (* p < 0.05; ** p < 0.01).

We next analyzed if the stimulation of the intrinsic NS2^pro^ activity by the F103A and L144I mutations is conserved among different HCV genotypes and whether a combination of both exchanges would further increase the intrinsic NS2^pro^ activity. To this end, we introduced these mutations individually and in combination into pcite-FLAG-NS2-APIT-GST/JFH1 (genotype 2a) and determined their impact on intrinsic NS2^pro^ activity side-by-side with their respective genotype 1b (BK) derivatives (Fig 5).

The comparison of the intrinsic NS2^pro^ activities of the two genotypes showed that the NS2 protease from the JFH1 strain (FLAG-NS2-NS3(1–172)-GST/JFH1-WT) achieved almost complete NS2-NS3(1–172) cleavage with no detectable FLAG-NS2-NS3(1–172)-GST/JFH1 precursor protein. The NS2-protease with a NS2-C184A mutation is deficient in supporting NS2-APIT cleavage in both genotypes, as expected. In contrast, the NS2 protease from the genotype 1b BK strain cleaved only approximately 70% of the FLAG-NS2-NS3(1–172)-GST/BK precursor protein (Fig 5A; compare FLAG-NS2-NS3(1–172)-GST/JFH1-WT with FLAG-NS2-NS3(1–172)-GST/BK-WT). This difference is in part attributable to a more efficient intrinsic NS2^pro^ activity (Fig 5A; compare FLAG-NS2-APIT-GST/JFH1-WT with FLAG-NS2-APIT-GST/BK-WT). The introduction of either NS2-F103A or NS2-L144I mutations resulted in a slightly increased intrinsic NS2^pro^ activity relative to the WT in the FLAG-NS2-APIT-GST context of both genotypes (Fig 5A; see FLAG-NS2-APIT-GST/JFH1-F103A and–L144I as well as FLAG-NS2-APIT-GST/BK-F103A and–L144I).

Interestingly, the combination of NS2-F103A and NS2-L144I resulted in a strong increase in NS2-APIT cleavage efficiency in both genotypes leading to an approx. 4-fold increase in cleavage efficiency compared to WT. In the context of genotype 2a, this F103A/L144I combination led to a remarkable efficient NS2-APIT cleavage of approx. 65% (Fig 5B). Together, this observation demonstrates that the stimulating effect of F103A/L144I amino acid exchanges on the intrinsic NS2^pro^ activity is conserved between different HCV genotypes (Fig 5). The ability of the double mutation F103A/L144I to strongly increase the intrinsic NS2^pro^ activity suggests that the functional roles of F103 and L144 might be mechanistically linked (Fig 5).

### The stimulating NS2 protease mutations F103A and L144I enhance the intrinsic NS2 proteolytic activity in absence of any NS3 sequence

**Fig 6:**
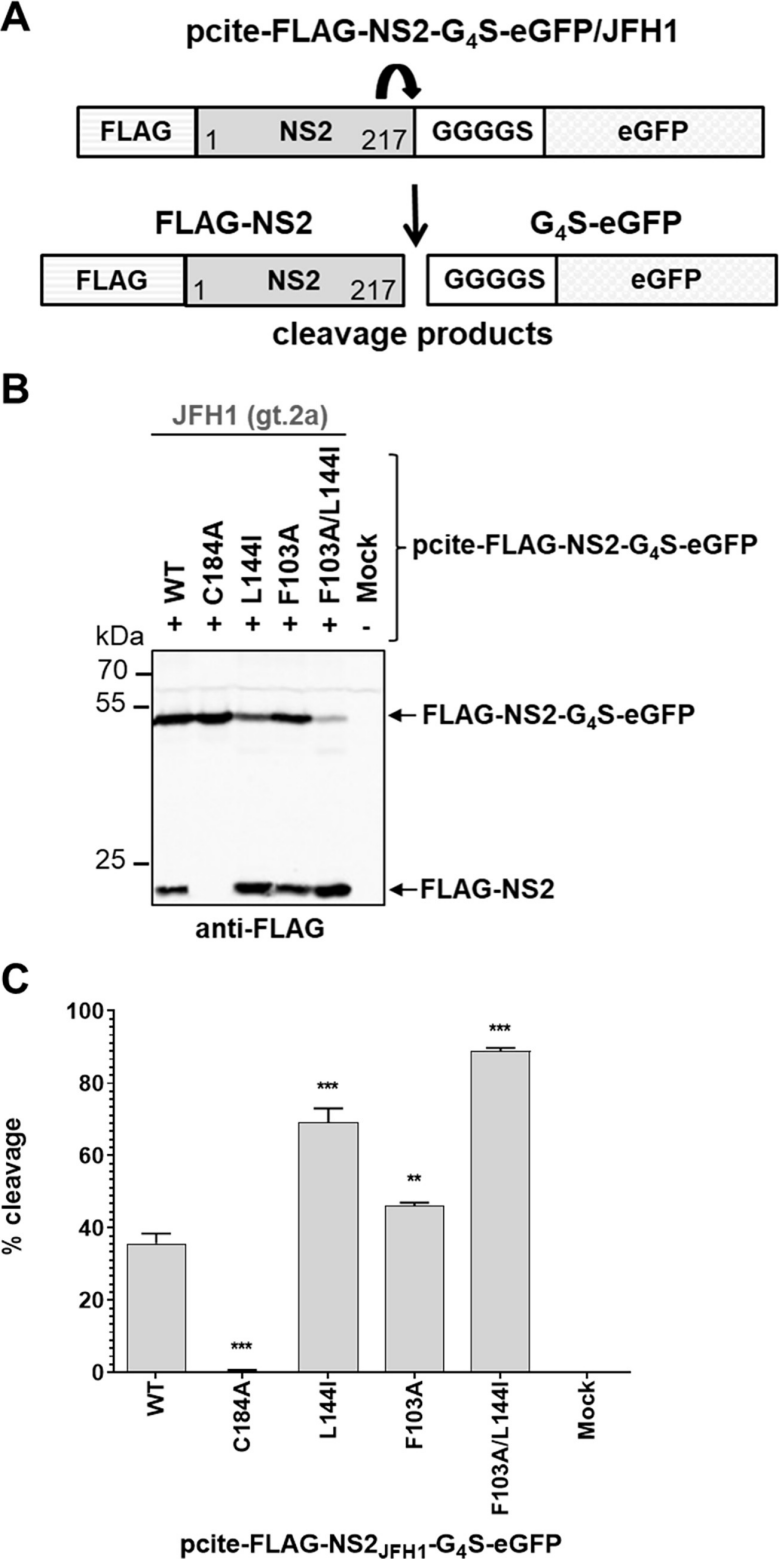
HCV NS2^pro^ activity in the absence of any NS3 sequence is stimulated by NS2 mutations F103A and L144I. (A) Scheme of pcite-FLAG-NS2_JFH1_-G_4_S-eGFP reporter construct to analyze NS2^pro^ activity in the absence of NS3 cofactor domain in the context of a flexible linker (GGGGS). (B) The indicated plasmids were transfected into MVA/T7^pol^-infected Huh7-T7 cells. Transfected cells were harvested and NS2^pro^ activities were analyzed by Western blot using anti-FLAG specific primary antibodies. One representative blot from three independent experiments is shown. The positions of the uncleaved precursor FLAG-NS2_JFH1_-G_4_S-eGFP and the cleavage product FLAG-NS2 are indicated by arrows on the right. (C) NS2 protease cleavage efficiencies. Western blot signals generated by using anti-FLAG-specific primary and infrared fluorescent-labeled secondary antibodies were captured by the Odyssey SA imaging system (LI-COR). Mean and standard deviations of NS2 cleavage efficiencies from three independent experiments are presented. WT: wild-type; mock: transfection control without DNA. Asterisks indicate statistically significant differences in comparison to WT (* p < 0.05; ** p < 0.01; *** p < 0.001).

It was previously reported that HCV NS2 exhibit efficient intrinsic activity in the absence of any NS3 sequence and that NS3-specific amino acids immediately downstream of NS2 (NS3-APITA) are reducing NS2^pro^ function to a basal level that requires NS3 cofactor-mediated stimulation for efficient NS2-NS3 cleavage [11]. Based on these observations, it was suggested that the NS3 N-terminal sequence is having a negative modulating impact on NS2-NS3 cleavage that is compensated by specific surface residues in the NS3 protease domain, resulting in NS2^pro^ stimulation by NS3 [11,13]. To determine if the stimulating effect of the F103A and L144I mutations on the NS2^pro^ activity in the pcite-FLAG-NS2-APIT-GST context is compensating for the negative impact of the NS3 N-terminal APIT sequence, we analyzed if the NS2^pro^ activity is stimulated by the F103A and L144 mutations in the absence of any NS3 sequence. We inserted both mutations into the pcite-FLAG-NS2_JFH1_-G_4_S-eGFP construct (Fig 6A). In this construct FLAG-NS2 is followed by the amino acid linker sequence GGGGS and eGFP so that the NS3 sequence downstream of NS2 is replaced by an unrelated sequence. Thus, we determined the effect of the stimulating amino acid exchanges F103A, L144I or F103A/L144I in the context of FLAG-NS2_JFH1_-G_4_S-eGFP in the absence of any NS3 sequence (Fig 6B and 6C). The C184A mutant served as negative control.

We detected robust NS2^pro^ cleavage activity (approx. 35%) with the FLAG-NS2_JFH1_-G_4_S-eGFP reporter construct confirming earlier observations [11]. Furthermore, the stimulating character of the NS2-F103A and NS2-L144I amino acid exchanges was also observed in absence of any NS3 sequence. Interestingly, the NS2-F103A mutation increased NS2^pro^ cleavage efficiency in the NS2_JFH1_-G_4_S-eGFP context only slightly from 35% to approx. 50%, while the introduction of NS2-L144I amino acid exchange resulted in a higher improvement (approx. 70%) of the intrinsic NS2^pro^ activity (Fig 6B and 6C). Importantly, the NS2-F103A/L144I combination led to approximately 90% NS2^pro^ cleavage efficiency. Together, the stimulation of the intrinsic NS2^pro^ activity observed for the FLAG-NS2-APIT-GST_F103A/L144I as well as for the NS2_JFH1_-G_4_S-eGFP_F103A/L144I variant is indicating that the identity of the residues C-terminal to the scissile bond are not contributing to the increase of the intrinsic NS2^pro^ activity. An attractive hypothesis is that the F103A/L144I exchanges contribute to an optimized NS2^pro^ active site geometry that leads to an enhanced NS2^pro^ activity.

### The L144I mutation is activating the intrinsic NS2 protease activity in other mammalian hepaciviruses

**Fig 7:**
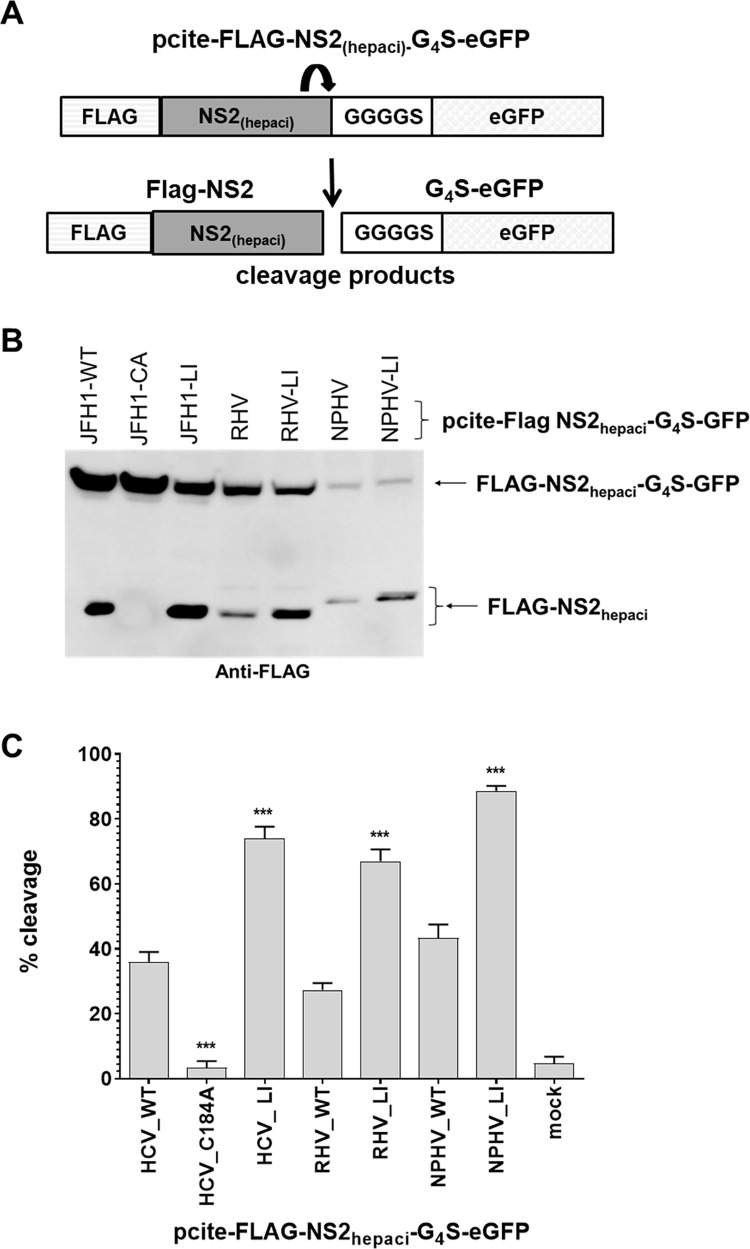
Leucine-to-isoleucine exchanges in other hepaciviral NS2 proteins corresponding to mutation HCV NS2 L144I are activating the intrinsic NS2^pro^ activity. (A) Scheme of pcite-FLAG-NS2_hepaci_-G_4_S-eGFP reporter construct to analyze the intrinsic NS2 protease activity of related mammalian hepaciviruses. (B) Indicated pcite plasmid derivatives (pcite-FLAG-NS2_hepaci_-G_4_S-eGFP) were transfected into MVA/T7^pol^-infected Huh7-T7 cells. Transfected cells were harvested in lysate buffer and NS2^pro^ activities were analyzed by Western blot analysis using anti-FLAG-antibodies. One representative blot out of three independent experiments is shown. Molecular mass standards are depicted on the left. The positions of the uncleaved precursors FLAG-NS2_hepaci_-G_4_S-eGFP or FLAG-NS2_hepaci_ cleavage product are indicated by arrows on the right. (C) NS2^pro^ cleavage efficiencies (indicated as percent of cleavage determined by percent of cleavage product over the sum of uncleaved precursor plus cleaved product) determined from lysates subjected to immunoblotting using infrared fluorescent-labeled secondary antibodies and the Odyssey SA imaging system (LI-COR). Mean and standard deviations of NS2^pro^ cleavage efficiencies from three independent experiments are presented. Asterisks indicate statistically significant differences in comparison to the respective WT (* p < 0.05; ** p < 0.01; *** p < 0.001).

Comparative studies of several mammalian hepaciviruses have been useful to identify conserved or divergent features of NS2 protein function(s) [11,14]. Alignment of NS2 sequences of different mammalian hepaciviruses revealed that residues corresponding to HCV NS2 L144 are conserved among these viruses suggesting a functional importance for this residue (Fig 4C; [11]). Therefore, we investigated whether the ability of the L144I exchange to activate the intrinsic NS2^pro^ activity is also conserved. For this purpose, we selected two hepaciviral NS2 proteins: equine hepacivirus (NPHV) strain H3-011 (NS2_NPHV_) closely related to HCV and the rodent hepacivirus (RHV) strain NLR07-oct70 (NS2_RHV_) which is more distantly related to HCV. We generated constructs pcite-FLAG-NS2_RHV_-G_4_S-eGFP and pcite-FLAG-NS2_NPHV_-4GS-eGFP (Fig 7A) and introduced the leucine-to-isoleucine mutation at positions corresponding to HCV NS2-L144 (e.g., NS2_NPHV_ L144I and NS2_RHV_ L132I).

The autoproteolytic properties of NS2_NPHV_-G_4_S-eGFP/L144I or NS2_RHV_-G_4_S-eGFP/L132I were compared with their respective wild-type constructs after transient expression in MVA/T7^pol^-infected Huh7-T7 cells. We used pcite-FLAG-NS2_HCV_-G_4_S-eGFP/WT and L144I from JFH1 strain of HCV genotype 2a as reference. Western blotting with FLAG-specific antibody was used to monitor NS2 protease activities. Interestingly, the intrinsic activity of both RHV and NPHV NS2 proteases could be stimulated by NS2_NPHV_ L144I and NS2_RHV_ L132I exchanges relative to their respective wild-type proteins, similar to what could be observed for NS2_HCV_ L144I (Fig 7B and 7C). Accordingly, these observations demonstrate that NS2_HCV_ L144 and corresponding residues in other mammalian hepaciviruses (NS2_NPHV_ L144 and NS2_RHV_ L132) represent a conserved functional determinant for the activation of the intrinsic NS2^pro^ activity in these viruses.

### Mutations stimulating NS2 autoprotease modulate viral RNA replication in a NS2-NS5B replicon

**Fig 8:**
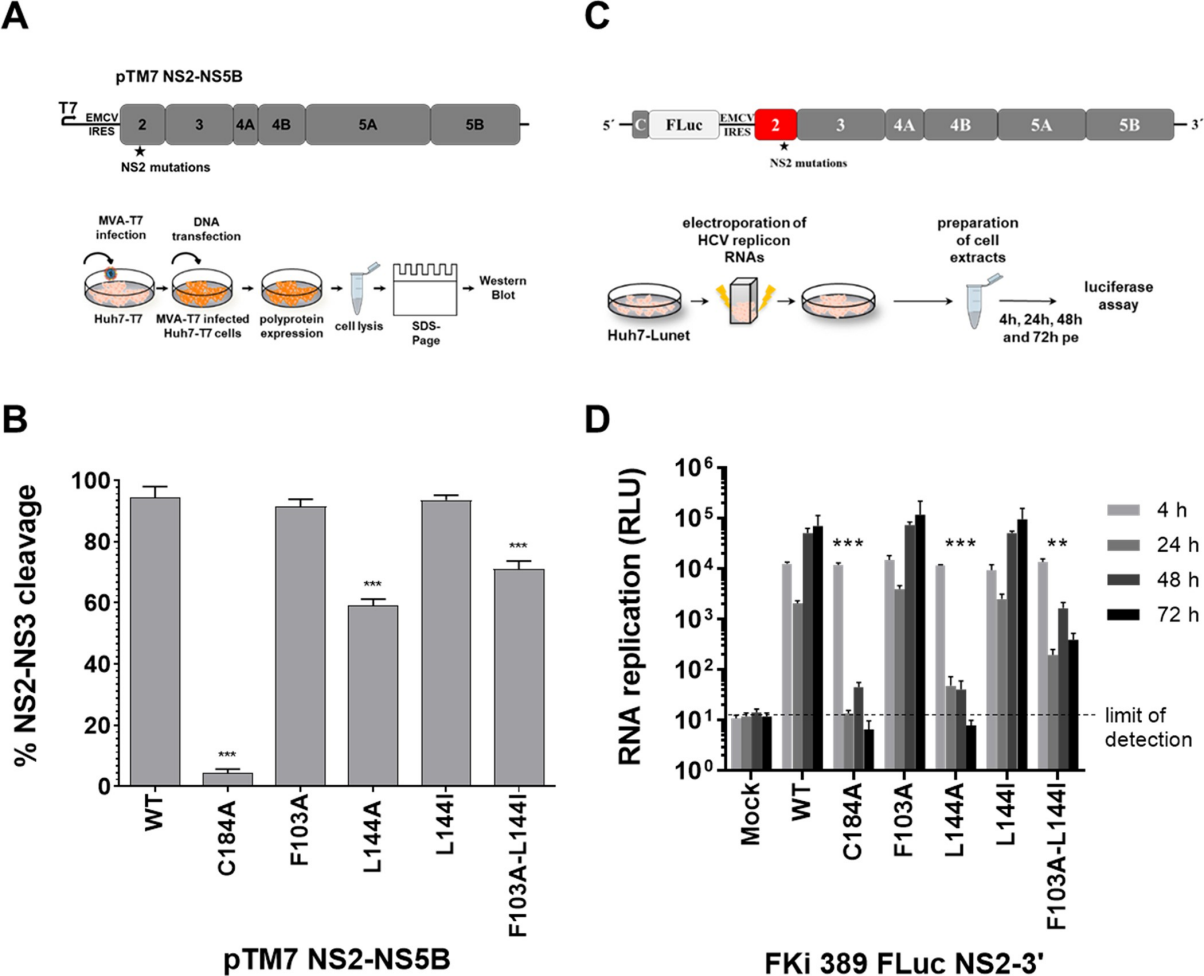
The NS2 F103A/L144I double mutation is reducing NS2-NS3 cleavage efficiencies in the NS2-NS5B context and is reducing robust RNA replication of a NS2-5B JFH1 replicon. (A, upper panel) Scheme of pTM7-NS2-NS5B/JFH1. (A, lower panel) schematic of the NS2-NS3 cleavage assay. 6 μg of the indicated pTM7-NS2-NS5B/JFH1 plasmids were transfected into MVA/T7^pol^-infected Huh7-T7 cells. Lysates were analyzed by SDS/PAGE-Western blotting using HCV anti-NS2 and anti-NS3 antibodies, respectively. (B) Quantification of the NS2-NS3 cleavage. Western blot signals of NS2, NS3 and uncleaved NS2-NS3 of three independent Western blots were quantified by ImageJ software and the percentage of NS2-NS3 cleavage (% NS2-NS3 cleavage) was calculated. Mean and standard deviations of NS2-NS3 cleavage efficiencies are presented. (C, upper panel) Experimental setup for RNA replication assay. Huh7 Lunet cells were electroporated with 10 μg of respective FKi-389_FLuc-NS2-3’/JFH1 RNAs. Lower panel, determination of viral replication. HCV RNA replication kinetics were quantified at 4, 24, 48 and 72 h post electroporation by measuring firefly luciferase activity (relative light units, RLU). Graphs are representative results of three independent experiments with standard deviations. WT: wild-type; mock: electroporation control without RNA. Asterisks indicate statistically significant differences in comparison to WT (* p < 0.05; ** p < 0.01; *** p < 0.001).

The observation that the NS2 protease has efficient intrinsic proteolytic activity in the absence of the NS3 cofactor is suggesting NS3 protease domain acts as a more complex, regulatory cofactor for NS2 to control the critical NS2-NS3 cleavage (Fig 6; [11]). To determine whether the presence of the activating NS2 mutations F103A and L144I is also stimulating NS2-NS3 cleavage in the HCV polyprotein context and thus enhancing RNA replication, we investigated their effect in the pTM7-NS2-NS5B polyprotein expression construct and in the pFki389 FLuc_NS2-5B/JFH1 replicon (Fig 8A and 8C).

Accordingly, we introduced the NS2 mutations F103A, L144A, L144I and F103A-L144I into pTM7-NS2-NS5B/JFH1 and pFki389 FLuc_NS2-5B plasmids (Fig 8A and 8C). The NS2 inactivating C184A mutation served as negative control in all experiments. To determine NS2-NS3 cleavage, the indicated pTM7-NS2-NS5B/JFH1 plasmids were transfected into MVA/T7^pol^-infected Huh7-T7 cells. This setup allows the replication-independent expression of the NS2-5B polyprotein to determine NS2-NS3 cleavage efficiency by Western blotting (Fig 8A and 8B). As expected, the Western blot analysis showed that WT NS2 supported efficient NS2-NS3 cleavage, while NS2 active site mutation C184A blocked NS2-NS3 cleavage (Fig 8B). The individual activating NS2 exchanges F103A and L144I exhibited NS2-NS3 cleavage efficiencies comparable to WT while the NS2 L144A mutation reduced NS2-NS3 cleavage (Fig 8B). These results were confirmed using pcite-FLAG-NS2-NS3(1–172)-GST/JFH1 and pcite-FLAG-NS2-NS3(1–172)-GST/BK, respectively (S1 Fig).

Interestingly, the simultaneous introduction of both stimulating NS2 exchanges (F103A/L144I) in the context of the NS2-NS5B polyprotein reduced the NS2-NS3 cleavage compared to WT and the F103A and L144I single mutations (Fig 8B). Accordingly, the F103A/L144I exchanges appear to interfere with an efficient NS2-NS3 cleavage in a NS2-NS5B polyprotein. This is in contrast to their stimulating effect on the intrinsic NS2^pro^ activity in the absence of its activating NS3^pro^ domain (compare Fig 8B with Figs 5 and 6). These observations are indicating that interactions between previously identified specific activating NS3^pro^ residues and a NS2^pro^ surface area comprising these NS2 mutations could contribute to an optimized NS2^pro^ active site conformation and efficient NS2-NS3 cleavage.

Next, RNA replication capabilities of the bicistronic Fki389 FLuc_NS2-NS5B/JFH1 derivatives were analyzed (Fig 8C and 8D). Replicon RNAs were *in vitro*-transcribed from *Mlu*I-linearized Fki389 FLuc_NS2-NS5B/JFH1 template DNAs and electroporated into Huh7 Lunet cells. The Fki389 FLuc_NS2-NS5B/JFH1_C184A plasmid with a C184A mutation served as negative control. RNA replication of the Fki389 FLuc_NS2-NS5B/JFH1 replicon derivatives with mutations F103A or L144I was similar to WT (Fig 8D) in agreement with their efficient NS2-NS3 cleavage in the pTM7-NS2-NS5B/JFH1 context (Fig 8B). In contrast, replication of the Fki389 FLuc_NS2-NS5B/JFH1_L144A replicon RNA was blocked (Fig 8D), which correlates with its reduced NS2-NS3 cleavage efficiency (Fig 8B). RNA replication of the Fki389 FLuc_NS2-NS5B/JFH1_F103A/L144I replicon RNA was approx. 50-150-fold lower compared to WT, F103A or L144I replicon RNAs at 48 h and 72 h pe (Fig 8D). These data confirm that the F103A/L144I double exchange, which allows for enhanced intrinsic NS2^pro^ activity in the absence of the NS3 cofactor, is reducing NS2-NS3 cleavage efficiency in the NS2-NS5B polyprotein context as well as RNA replication efficiency. Together these observations indicate that optimal surface interactions between NS2 and NS3 are pivotal for efficient NS2-NS3 cleavage and thus critical for viral RNA replication.

### Evidence for a functional cooperation of NS2 and NS3 surface residues during NS3-mediated NS2 protease stimulation

While NS2_HCV_ L144 and corresponding residues in other mammalian hepaciviruses (NS2_NPHV_ L144 and NS2_RHV_ L132) are conserved and functionally important for the activation of the intrinsic NS2^pro^ activity in these viruses, the residues corresponding to NS2_HCV_ F103 show no such conservation (Fig 4C).

It has been demonstrated that the NS2_HCV_ protease activity could be stimulated by heterologous hepacivirus NS3 protease domains from NPHV, BHV and RHV but only very weakly from GHV and GBV-B, indicating specific requirements for the activating residues within the hydrophobic NS3 surface patch [11]. This ability of NS3_GHV_ to activate NS2_HCV_ protease in the context of NS2_HCV_-NS3_GHV_ chimera was shown to rest on hydrophobic NS3 surface residues [11]. Interestingly, in the activating NS3 of NPHV, BHV and RHV conserved proline residues are present at NS3 positions equivalent to NS3_HCV_ Pro115 whereas in the non-activating GHV NS3 protease domains NS3_HCV_ Pro115 aligns with a negatively charged glutamate residue (NS3_GHV_ E116). When NS3_GHV_ E116 was changed into the HCV-specific Pro (NS3_GHV_ E116P) a substantial gain-of-function of the NS3_GHV_ protease domain as stimulating cofactor for HCV NS2 protease in the chimeric NS2_HCV_-NS3_GHV_ precursor was observed [11]. Accordingly, this chimeric NS2_HCV_-NS3_GHV_ precursor represents an excellent tool to study potential NS2-NS3 surface interactions during NS3-mediated NS2 protease stimulation.

**Fig 9:**
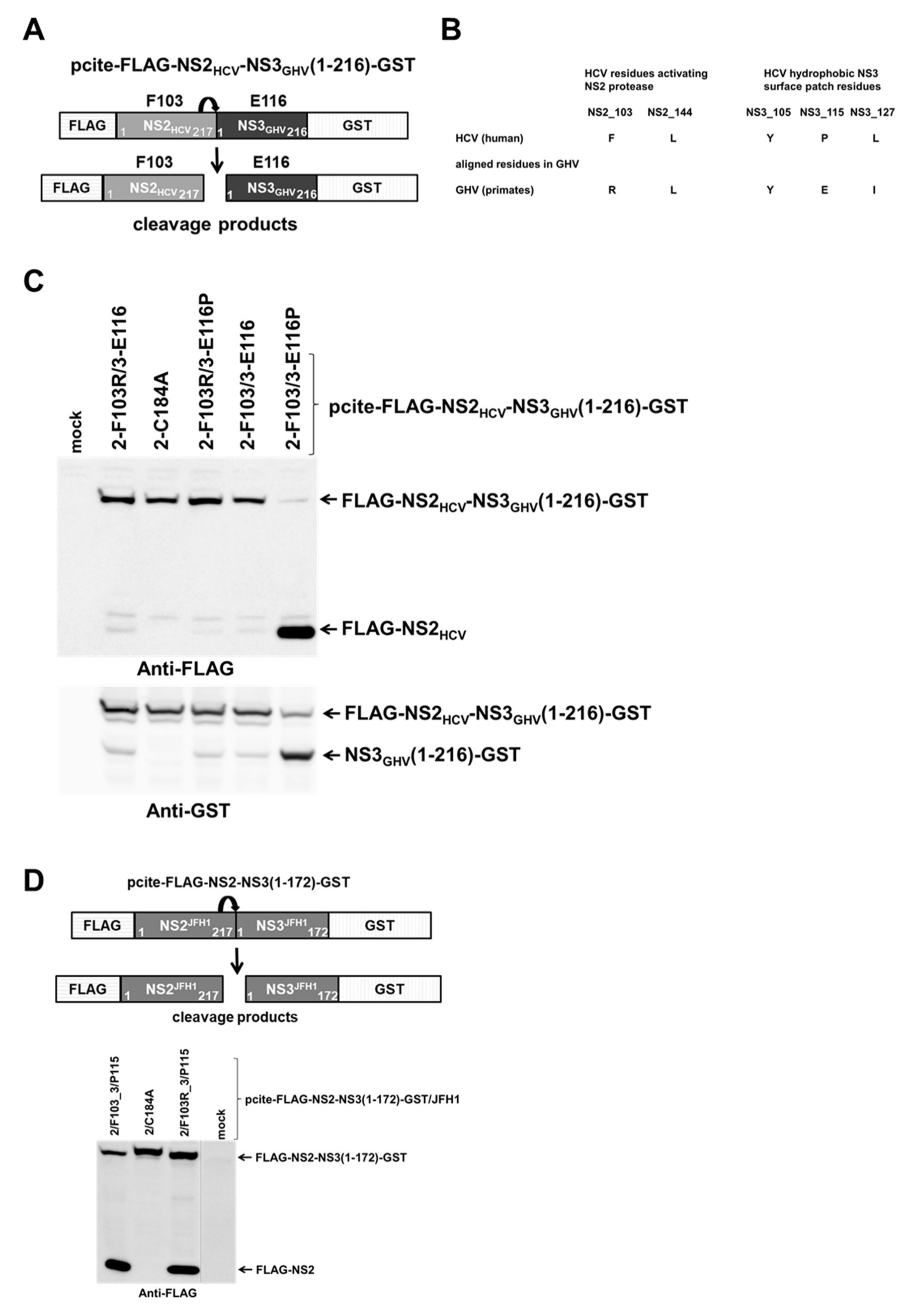
HCV-specific surface residues at the proposed NS2-NS3 interaction interface of a chimeric NS2_HCV_-NS3_GHV_ precursor are required for efficient NS2-NS3 cleavage. (A) Scheme of pcite-FLAG-NS2_HCV_NS3_GHV_-GST reporter construct to analyze NS2-NS3 cleavage in a chimeric FLAG-NS2_HCV_-NS3_GHV_-GST precursor protein. (B) GHV-specific residues corresponding to HCV NS2 (F103- and L144) and NS3 (Y105, P115, L127) amino acids important for NS3-mediated NS2^pro^ activation [11]. (C) Cleavage of pcite-FLAG-NS2_HCV_NS3_GHV_-GST derivatives. (D) Upper, scheme of pcite-FLAG-NS2_JFH1_-NS3_JFH1_-GST reporter. (C and D lower panel), indicated plasmids were transfected into MVA/T7^pol^-infected Huh7-T7 cells. Transfected cells were harvested and NS2-NS3 cleavage was monitored by Western blot analysis using anti-FLAG and anti-GST antibodies. One representative blot out of three independent experiments is shown. Molecular mass standards are depicted on the left. The positions of the uncleaved precursors FLAG-NS2_HCV_-NS3_GHV_(1–216)GST or FLAG-NS2-NS3(1–172)-GST and cleaved products FLAG-NS2_HCV_, NS3_GHV_-(1–216)-GST are indicated by arrows on the right. WT: wild type; mock: transfection control without DNA.

We generated a similar chimeric FLAG-NS2_HCV_-NS3_GHV_-GST precursor with an N-terminal FLAG and C-terminal GST tag (Fig 9A) to determine whether NS2_HCV_ F103 is interacting with NS3_GHV_ surface residues during NS3_GHV_-mediated NS2_HCV_ protease stimulation. We hypothesized that NS2_HCV_ F103 has to interact with NS3_GHV_ E116 (equivalent to NS3_HCV_ P115, Fig 9B) during NS2_HCV_ activation by NS3_GHV_. This assumption was based on two observations: (i) in our NS2-NS3 precursor model NS2 F103 has the potential to interact with NS3 P115 (Fig 1A) and (ii) mutating NS3_GHV_ E116 to proline (NS3_GHV_ E116P) rescues the inefficient NS2_HCV_ activation by heterologous NS3_GHV_ [11].

To test if the surface residues NS2_HCV_-F103 and NS3_GHV_ E116 functionally interact during NS2_HCV_ activation by NS3_GHV_, we determined the NS2-NS3 cleavage of different chimeric FLAG-NS2_HCV_-NS3_GHV_-GST precursor variants. Accordingly, we generated pcite-FLAG-NS2_HCV_-NS3_GHV_-GST variants with a GHV-specific (2-F103R/3-E116) or a HCV-specific (2-F103/3-E116P) surface residue pair. In addition, we analyzed the chimeric HCV/GHV (2-F103/3-E116) and GHV/HCV (2-F103R/3-P116) situations at these surface positions in the pcite-FLAG-NS2_HCV_-NS3_GHV_-GST context. An active-site mutation (2-C184A) was used as negative control. As reported previously [11], we also detected a weak NS2^pro^ activity in the chimeric context of FLAG-NS2_HCV_-NS3_GHV_-GST (2-F103/3-E116) indicating inefficient NS2_HCV_ protease stimulation by the heterologous NS3_GHV_ protease domain and the dramatic gain-of-function stimulation by introducing the HCV-specific proline into NS3_GHV_ (3-E116P) in the FLAG-NS2_HCV_-NS3_GHV_-GST (2-F103/3-E116P) (Fig 9C). Importantly, this remarkable gain-of-function was blocked by replacing the NS2_HCV_-specific F103 with the GHV-equivalent arginine (2-F103R) resulting in very weak NS2-NS3 cleavage of this FLAG-NS2_HCV_-NS3_GHV_-GST (2-F103R/3-E116P) variant. This activity is similar to the one of the parental chimeric FLAG-NS2_HCV_-NS3_GHV_-GST (NS2_HCV_-F103/NS3_GHV_-E116) derivative indicating a functional incompatibility of NS2_HCV_-F103R with NS3_GHV_-E116P. This defect caused by the NS2_HCV_ F103R mutation is not due to defects within the NS2_HCV_ protease domain since a F103R mutation in the non-chimeric HCV FLAG-NS2-NS3 (1–172)-GST context supports NS2-NS3 cleavage comparable to wild-type (Fig 9D). The weak NS2-NS3 cleavage of the chimeric FLAG-NS2_HCV_-NS3_GHV_-GST could not be rescued by the combining GHV-specific hydrophilic residues (2-F103R/3-E116) (Fig 9C). Together, the results suggest that NS2_HCV_-F103 is indeed an important determinant of the NS2 stimulation by NS3. Furthermore, these data indicate that an optimal communication between both surface areas (surrounding F103/L144 in NS2_HCV_ and E116 in NS3_GHV_) is pivotal for efficient NS3-mediated NS2^pro^ stimulation in this FLAG-NS2_HCV_-NS3_GHV_-GST chimera.

## Discussion

Intramolecular protein-protein interactions between NS2 and NS3 are proposed to play an essential role during NS2-NS3 cleavage due to the pivotal function of a conserved hydrophobic NS3 protease surface patch during NS3-mediated NS2^pro^ stimulation [11–13]. Despite recent insights into NS2^pro^ stimulation by NS3, including the recent findings that NS3^pro^ domain acts as a complex, regulatory cofactor for hepaciviral NS2 proteases, many mechanistic details of this protease activation are still unknown [11,13]. Especially, the interactions between these specific NS3 residues and a yet unidentified region on the NS2^pro^ surface that are proposed to contribute to NS2^pro^ active site conformation in hepaciviruses remained enigmatic.

Among the tested NS2 mutations, which were chosen based on a *in silico* NS2-NS3 precursor model, only L144A and L147A mutations phenocopied the inhibitory effect of the NS3 Y105A/P115A/L127A (YPL-AAA) surface mutations during NS3-mediated NS2^pro^ activation (Fig 1) [13]. The residues L144 and L147 are highly conserved and are close to the NS2^pro^ active site residue H143 suggesting they have a critical function. In the case of the L144, a functional importance in NS2-3 processing and RNA replication has been already observed [25]. Specifically, this residue was suggested to assist in the formation of the correct NS2^pro^ active site architecture [25]. Interestingly, in the absence of the activating NS3 cofactor we detected differences between L144A and L147A mutations with regard to their impact on the intrinsic NS2^pro^ activity (Fig 2). While the L144A variant allowed wild-type-like intrinsic NS2^pro^ activity in the FLAG-NS2-APIT-GST context, the L147A mutation inhibited this activity (Fig 2). From these results we conclude that the decrease for the FLAG-NS2-NS3(1–172)-GST L147A mutant during NS3-mediated NS2^pro^ stimulation is most likely due to its interference with optimal NS2^pro^ active-site formation and/or local NS2^pro^ protein folding. Together, these observations indicate that the NS2 L144 surface residue functions as a potential NS2 determinant during NS2^pro^ stimulation by NS3.

Another surprising aspect of this study was the identification of two amino acid exchanges (F103A and L144I) that stimulated the intrinsic NS2^pro^ activity in the absence of NS3 (Figs 2 and 3). Importantly, we found that a combination of F103A and L144I further stimulates the intrinsic NS2^pro^ activity, suggesting both exchanges might complement each other. Interestingly, our permutation analysis revealed striking differences of the amino acid requirements at both positions: While only L144I was clearly enhancing the intrinsic NS2^pro^ activity, several F103 amino acid exchanges (e.g., F103A, F103Q, F103R and F103N) were able to stimulate the intrinsic NS2^pro^ activity (Figs 3 and 4 and 9D). One possible interpretation of these observations is that the ability of these exchanges to stimulate the intrinsic NS2^pro^ activity might be achieved through distinct mechanisms (Figs 3 and 4). Importantly, the ability of F103A and L144I to stimulate intrinsic NS2^pro^ activity was confirmed in different HCV genotypes regardless of genotype-specific differences in their intrinsic cleavage efficiencies of FLAG-NS2-APIT-GST/JFH1 (genotype 2a) compared to FLAG-NS2-APIT-GST/BK (genotype 1b) (Fig 5). This functional conservation clearly supports their importance for the stimulation of the intrinsic NS2^pro^ activity.

The identification of amino acid exchanges that increase the intrinsic NS2^pro^ activity above wild-type level could point to changes within NS2^pro^ domain required for optimized NS2 active-site formation and/or improved positioning of the cleavage site relative to the protease active-site. Of note, such changes within NS2 in the context of the viral polyprotein most likely are induced by selected surface residues in NS3^pro^ domain, whose translation and folding are promoting NS2^pro^ stimulation [11,13]. Thus, an attractive hypothesis is that these stimulating amino acid exchanges are providing first insights into the molecular dynamics within NS2 during NS2^pro^ activation.

How exactly these stimulating NS2 exchanges modulate NS2^pro^ activity remains to be determined. Mechanistically, the NS2^pro^ stimulation by its cofactor NS3 could involve optimization of the active site conformation, a more efficient global protein folding and/or a better positioning of the NS2-NS3 cleavage site relative to the nucleophilic active site cysteine residue. Along these lines, it is noteworthy that the F103A/L144I exchange is increasing the intrinsic NS2^pro^ activity not only in the FLAG-NS2-APIT-GST but also in the FLAG-NS2-GS_4_-eGFP context that has downstream of NS2 no sequence similarity to HCV NS2-NS3 cleavage sites (Figs 4 and 5). Therefore, we currently favor the hypothesis that these NS2 mutations stimulate NS2 protease function by optimizing protease active site geometry and/or by increasing local protein folding in a conserved fashion rather than by better positioning of the NS2-NS3 cleavage site relative to the NS2 active-site. Interestingly, a possible contribution to the establishment of the correct geometry of the NS2^pro^ active site was proposed for P164 which is in close proximity to the catalytic E163 residue, most likely *via* its *cis*-conformation [8]. Of note, the NS2-NS3 cleavage reaction is bimolecular [26,27], and the dimeric enzymatic mechanism of NS2^pro^ was experimentally confirmed not only for HCV but also for related mammalian hepaciviruses [8,11].

The strict conservation of NS2 L144 in HCV genotypes and in other mammalian hepaciviruses at equivalent positions argues for a central role of this leucine during NS3-mediated NS2^pro^ stimulation. In support of this assumption, we observed an enhancement of the intrinsic NS2^pro^ activities in related hepacivirus NS2 variants (e.g., NS2_RHV_ L132I and NS2_NPHV_ L144I) with leucine-to-isoleucine exchanges at NS2 positions equivalent to NS2_HCV_ L144 (Fig 8). A possible explanation for these observations could be that the replacement of leucine with isoleucine at these positions leads to local changes around the active-site histidine that potentially optimizes the NS2^pro^_NPHV_ and NS2^pro^_RHV_ active-site geometry in the absence of NS3 cofactor. Interestingly, for HCV NS2 it has been shown that H143 makes a higher number of hydrogen bond contacts when compared to C184 [8]. The importance of these hydrogen bond contacts for NS2 protease function was supported by the observation that a H143A, but not a C184A mutation leads to a global loss of NS2 secondary structure characteristics [28].

The fact that NS2 dimerization is required for proteolysis led to the hypothesis that a certain level of NS2 has to accumulate before NS2-NS3 processing and initiation of RNA replication can occur efficiently. Accordingly, an increased intrinsic NS2^pro^ activity of the NS2 F103A/L144I variant should lead to increased viral replication. However, the introduction of these mutations into a NS2-NS5B RNA replicon reduced its RNA replication capacity (Fig 7D). The reduced efficiency of the NS2-NS3 cleavage observed for this mutant in the context of the NS2-NS5B polyprotein most likely caused this attenuated replication phenotype (Fig 7C). This observation suggests a F103A/L144I-mutated NS2 exhibits a reduced ability to interact with stimulating NS3 surface residues critical for efficient NS2-NS3 cleavage. Such inefficient NS2/NS3 communication within uncleaved NS2-NS3 is expected to decrease the kinetics of replication initiation, which relies on NS2-NS3 cleavage for the release of NS3-NS4A to drive the active formation of the membranous HCV RC and therefore would delay or inhibit viral replication [29]. Such a delay of RNA replication initiation may also be required for the virus to produce sufficient NS3/4A to antagonize cellular antiviral signaling before the onset of viral replication [30].

Mechanistically, the NS2 F103 surface residue is the prime candidate for an amino acid that contributes to the NS3-mediated NS2^pro^ activation *via* NS2:NS3 surface contacts. Support for this hypothesis comes from experiments with chimeric NS2_HCV_-NS3_GHV_. Its weak NS2_HCV_-NS3_GHV_ cleavage was proposed as an indication for a functional incompatibility between interacting NS2 and NS3 surface areas during NS3-mediated NS2^pro^ activation ([11] and Fig 9). It was shown that this incompatibility is eliminated by a NS3_GHV_ E116P exchange within the activating NS3 surface patch that strongly increased the NS2^pro^ stimulation by NS3_GHV_ ([11] and Fig 9). Thus, the NS3_GHV_ E116P exchange most likely creates a specific NS3_GHV_ surface area better suited to interact with the NS2_HCV_ surface around NS2 F103. Supporting this assumption is our observation that a combination of NS2_HCV_ F103R mutation with a NS3_GHV_ E116P exchange in NS2_HCV_-NS3_GHV_ E116P context was drastically reducing the strong NS2_HCV_ protease activation by the NS3_GHV_ E116P cofactor domain (Fig 9C) without affecting the NS2_HCV_ protease activity in the non-chimeric HCV FLAG-NS2-NS3(1–172)-GST context (Fig 9D). These observations suggest the NS2_HCV_ F103R mutation disrupts favorable surface interactions during NS2_HCV_ protease activation by the heterologous NS3_GHV_ E116P cofactor domain. It is noteworthy that the defect caused by the NS2_HCV_ F103R mutation could not be rescued by wild-type NS3_GHV_ with E116 in the chimeric NS2_HCV_-NS3_GHV_ context (Fig 9C). One possible explanation is that the formation of a proposed surface interaction between both residues with electrostatic complementarity was not sufficient to rescue the loss-of-function phenotype. Most likely, additional surface residues are involved and required for efficient NS2^pro^ stimulation in this chimeric context. Together, these results not only highlight the role of the hydrophobic NS3 surface patch for NS3 cofactor activity during NS2^pro^ stimulation but also provide first evidence for an involvement of a NS2 surface region involving F103 and L144 in this activation process.

## Materials and methods

### Cell culture

Huh7 Lunet [31], Huh7.5 and Huh7-T7 [32] cells were maintained in Dulbecco’s modified Eagle medium (DMEM) supplemented with 10% fetal calf serum (FCS), 100 U penicillin/100 μg/ml streptomycin, and 2 mM L-glutamine. Huh7-T7 cells were maintained in the presence of 400 μg/ml G418.

### Plasmids and mutagenesis

The HCV genomes BK [33] and JFH1 [34] have been described. All mutations were introduced *via* QuikChange (QC) mutagenesis according to instructions by the manufacturer (Thermo Scientific). Plasmids pcite-FLAG-NS2-NS3(1–172)-GST/BK and pcite-FLAG-NS2-NS3(1–172)-GST/JFH1have been described [13]. The plasmids pcite-FLAG-NS2-APIT-GST/JFH1 and pcite-FLAG-APIT-GST/BK were generated as follows: an second *Age*I restriction site was introduced into pcite-FLAG-NS2-NS3(1–172)-GST/JFH1 and pcite-FLAG-NS2-NS3(1–172)-GST/BK by QuikChange mutagenesis using primers APIT_JFH_-AgeI se (5’-ggggtggaagctccttgctcccatcactACCGGTgcccagcaaacacgaggcctcctg-3’) and APIT_JFH_-AgeI ase (5’-caggaggcctcgtgtttgctgggcACCGGTagtgatgggagcaaggagcttccacccc-3’) with pcite-FLAG-NS2-NS3(1–172)-GST/JFH1 as template or APIT_BK_-AgeI se (5’- Cgggggtggcgactcctc gcgcccatcacgACCGGTtcccaacagacgcggggcc -3’) and APIT_BK_-AgeI ase (5’- ggccccgcgtctgttgggaACCGGTcgtgatgggcgcgaggagtcgccacccccG -3’) with pcite-FLAG-NS2-NS3(1–172)-GST/BK as template. The resulting pcite_-FLAG-NS2-NS3(1–172)-GST/JFH1_AgeI and pcite-FLAG-NS2-NS3(1–172)-GST/BK_AgeI plasmids were cleaved with *Age*I and religated to generate pcite-FLAG-NS2-APIT-GST/JFH1 and pcite-FLAG-APIT-GST/BK, respectively.

The pCMV-NS2_RHV_-GGGGS-GFP, pCMV-NS2_NPHV_-GGGGS-GFP and pCMV-NS2_JFH1_-GGGGS-GFP constructs were a kind gift from Dr. Annette Martin (Institute Pasteur, Paris, France) and were used to generate pcite-FLAG-NS2_hepaci_-G_4_S-GFP derivatives. The pcite-FLAG-NS2_HCV_-G_4_S-GFP, pcite-FLAG-NS2_RHV_-G_4_S-GFP and pcite-FLAG-NS2_NPHV_-G_4_S-GFP constructs were generated as follows: NS2 sequences were amplified with NS2-specific primers; NS2_JFH1_-G_4_S: NS2_JFH1_ se (5’- CCCGGGtatgatgcccctgtgcacggccagatc-3’) and NS2_JFH1_ ase (5’- cacggatcctccgcctcccagcagcttccatcccttgC-3’); NS2_RHV_-G_4_S: NS2_RHV_ se (5’- CCCGGGagcctgaacgatgtgctgctggccgtgtcc-3’) and NS2_RHV_ ase (5’- cacggatcctccgcctccggttctttcccagccctgtG-3’); NS2_NPHV_-G_4_S: NS2_NPHV_ se (5’- CCCGGGttcgacaatacctctgccgtgaccgccgcc-3’) and NS2_NPHV_ ase (5’- cacggatcctccgcctcccagcagccgccatC-3’), respectively. PCR products were cloned into pGEM-T vector, resulting in pGEM-T NS2_JFH1_-G_4_S, pGEM-T NS2_RHV_-G_4_S and pGEM-T NS2_NPHV_-G_4_S, respectively. The NS2_JFH1_-G_4_S, NS2_RHV_-G_4_S and NS2_NPHV_-G_4_S inserts were obtained by cleaving the respective pGEM-T NS2_hepaci_-G_4_S plasmids with *Xma*I and *BamH*I, respectively. The GGGS-GFP fragment was taken from pCMV-NS2_RHV_-GGGGS-GFP by restriction with *BamHI* and *XbaI*. The pcite-FLAG-NS2_hepaci_- G_4_S-GFP plasmids were generated by cloning the respective FLAG-NS2_hepaci_ fragments and GGGS-GFP *via XmaI*, *BamHI* and *XbaI*.

Mutagenesis of JFH1 NS2-NS3 cDNA was performed on a plit28-KpnI-NsiI/NS2-NS3/JFH1 subclone containing the NS2-NS3/JFH1 fragment from pFKI389Luc/NS2-3’_dg [13]. The internal *KpnI* site has been destroyed by a silent mutation with oligos NS2_JFH1_*KpnI* QC se (5’-gaagccatgattcaggagtgggtcccacccatgcaggtgcgcg-3’) NS2_JFH1_*KpnI* QC ase (5’-cgcgcacctgcatgggtgggacccactcctgaatcatggcttc-3’). All mutations were cloned into pFKI389Luc/NS2-3’_dg via *KpnI*/*NsiI*.

### In vitro transcription and electroporation of HCV RNAs

The experimental procedures used to generate *in vitro* transcripts from cloned HCV sequences and transfection of Huh7 lunet cells by electroporation have been described [13]. After electroporation, cells were immediately transferred to complete DMEM and seeded as required for the assay.

### Determination of RNA replication by Luciferase assay

At each time point indicated (4 h, 24 h, 48 h, and 72 h post electroporation, pe), cells were washed with PBS, scraped into 1 ml of PBS and collected by centrifugation. The cells were lysed in 40 μl of lysis buffer (PJK-GmbH, Kleinblittersdorf, Germany). 20 μl of the lysate was analyzed using the Beetle Juice luciferase assay system (PJK-GmbH, Kleinblittersdorf, Germany) and firefly luciferase activity was measured in a luminometer (Junior LB9509, Berthold).

### Vaccinia virus infection, DNA transfection and transient protein expression

The applied procedures have been described [35]. HCV nonstructural proteins were expressed from pcite_ plasmids. Briefly, 2 x 10^6^ Huh7/T7 cells were infected with MVA/T7^pol^ vaccinia virus [36] for 1 h at 37°C and subsequently transfected with 4 or 8 μg of plasmid DNA by using Metafectene reagent (Biontex Laboratories GmbH, Munich, Germany). Transfected cells were incubated for either 24 h or 48 h at 37°C for protein expression. Cells were harvested at indicated time points and subsequently lysed for SDS-PAGE and western blot analysis.

### SDS-PAGE and Western blotting

Proteins were separated in polyacrylamide-Tricine gels. After SDS-PAGE, proteins were transferred onto a nitrocellulose membrane (Pall, USA). The membrane was blocked with 5% (w/v) dried skim milk in phosphate-buffered saline with 0.05% (v/v) Tween 20 (Invitrogen). For antigen detection, mouse monoclonal antibody against NS3 of the JFH-1 isolate (4D11; [13]) or anti-NS3 (2E3) [37], anti-FLAG (Sigma), anti-GFP (Cell signalling) and anti-GST (GE Healthcare), antibodies were used in 2% (w/v) dried skim milk in phosphate-buffered saline with 0.05% (v/v) Tween 20. For primary antibody detection, horseradish peroxidase-conjugated species-specific secondary antibodies (Dianova) were used at a 1:3000 dilution and Western Lightning Chemiluminescence Reagent Plus (Perkin Elmer) was applied prior to imaging using a LI-COR imaging system (Biorad, Munich).

### Quantifications of Western blots

Quantifications of NS2 cleavage efficiencies were carried out using ImageJ 1.47t (NIH, Bethesda). The quantification of cleavage efficiencies was done by the determination of Western blot signal intensities for uncleaved polyprotein precursor and cleaved product to calculate the NS2 cleavage rate. The respective primary antibodies were either detected by IRDye 800CW-labeled secondary antibodies using the Odyssey SA imaging system (LI-COR) or by horseradish peroxidase-conjugated species-specific secondary antibodies (Dianova) using the LAS 4000 imaging system (Biorad, Munich) and Western Lightning Chemiluminescence Reagent Plus (Perkin Elmer).

### Construction of a NS2-NS3 precursor model

To construct the NS2-NS3 model, the software SybylX 1.2, the information from the NS2 protease domain crystal structure (residues 94–217, pdb id 2HD0, [8]) and the crystal structure of the NS3 protease domain complexed with a synthetic NS4A cofactor peptide (pdb id 1A1R, [24]) representing protein sequences from Hepatitis C virus genotype 1a (isolate H77) were used. The model was constructed as follows: the number of molecules in the asymmetric unit was reduced to the smallest functional unit. Subsequently, hydrogen atoms were added by software and the B-factors were replaced by charges according to the Gasteiger-Huckel model. Refinement was achieved by undertaking independent energetic minimizations of both proteins [38].

The following constrains were integrated into the model: The activating NS3 hydrophobic region is formed by the amino acids Ile3, Tyr105, Pro115 and Ile127 [13]. The NS2 and the NS3 proteins are covalently linked with a minimal interaction radius between both proteins. The NS2-NS3 cleavage site should less than 4Å away from the active site sulfur atom of Cys184. The structures of NS2 and NS3 are assumed to be identical to the determined post-cleavage crystal structure, e.g., the NS2 is present as an active dimer, the NS3 protein as an active monomer.

The NS2-NS3 model was constructed in several stages: (i) analysis of the NS2 hydrophobic regions near the C-terminus and placement of the activating hydrophobic region of the NS3 protein into proximity of the identified NS2 regions, (ii) change of the binding angles of the NS2 C terminus and the NS3 N terminus to optimize the distance between both atoms to establish the covalent bond of the NS2-NS3 precursor protein and (iii) energetic minimization of the NS2-NS3 precursor protein.

### Statistical analysis

Experiments were conducted in at least three biological replicates unless stated otherwise. Graphical representations were performed with GraphPad Prism versions 8. Unless stated differently, results are presented as the mean ± the standard deviation (SD) of three biological replicates. Statistical significance was determined using a Students t-test with SigmaPlot software (Systat Software GmbH, Düsseldorf, Germany). P values of <0.05 were considered significant.

The numerical data used in all figures are included in S1 Data.

## Supporting information

S1 Fig NS2-NS3 cleavage in the FLAG-NS2-NS3(1–172)-GST context is reduced in the presence of the NS2-L144I/F103A double mutations compared to the wild-type. MVA/T7^pol^-infected Huh7-T7 cells were transfected with 4 μg of the indicated pcite-Flag-NS-NS3(1–172)-GST/BK (A) or pcite-Flag-NS-NS3(1–172)-GST/JFH1 (B) plasmid derivatives. Transfected cells were harvested 20 h post-transfection into lysate buffer. Afterwards, protein lysates were separated by SDS-PAGE and analyzed by Western blot using anti-GST antibody. Molecular mass standards are indicated on the left. Position of the uncleaved precursor Flag-NS2-NS3(1–172)-GST and cleavage product NS3(1–172)-GST are indicated by arrows on the right. WT: wild-type; mock: transfection control without DNA.
(TIF)

S1 Data Excel spreadsheet containing, in separate sheets, the underlying numerical data for Figure panels 1E, 2C, 3B, 4B, 5B, 6C, 7C, 8B, 8C. (XLSX)

## References

[1] Manns MP, Buti M, Gane E, Pawlotsky JM, Razavi H, Terrault N, et al. Hepatitis C virus infection. Nature reviews Disease primers. 2017;3:17006. doi: 10.1038/nrdp.2017.6 . 28252637

[2] Hartlage AS, Cullen JM, Kapoor A. The Strange, Expanding World of Animal Hepaciviruses. Annual review of virology. 2016;3(1):53–75. doi: 10.1146/annurev-virology-100114-055104 ; PubMed Central PMCID: PMC5523456. 27741408 PMC5523456

[3] Shimotohno K, Tanji Y, Hirowatari Y, Komoda Y, Kato N, Hijikata M. Processing of the hepatitis C virus precursor protein. Journal of hepatology. 1995;22(1 Suppl):87–92. . 7602084

[4] Lin C, Lindenbach BD, Prágal BM, McCourt DW, Rice CM. Processing in the Hepatitis C Virus E2-NS2 region: identification of p7 and two distinct E2- specific products with different C termini. J Virol. 1994;68:5063–73. doi: 10.1128/JVI.68.8.5063-5073.1994 7518529 PMC236449

[5] Grakoui A, McCourt DW, Wychowski C, Feinstone SM, Rice C. A second hepatitis C virus-encoded proteinase. Proc Natl Acad Sci USA. 1993;90:10583–7. doi: 10.1073/pnas.90.22.10583 8248148 PMC47821

[6] Hijikata M, Mizushima H, Akagi T, Mori S, Kakiuchi N, Kato N, et al. Two distinct proteinase activities required for the processing of a putative nonstructural precursor protein of hepatitis C virus. J Virol. 1993;67(8):4665–75. Epub 1993/08/01. doi: 10.1128/JVI.67.8.4665-4675.1993 . 8392606 PMC237852

[7] Failla C, Tomei L, de Francesco R. Both NS3 and NS4A are required for proteolytic processing of hepatitis C virus nonstructural proteins. J Virol. 1994;68:3753–60. doi: 10.1128/JVI.68.6.3753-3760.1994 8189513 PMC236880

[8] Lorenz IC, Marcotrigiano J, Dentzer TG, Rice CM. Structure of the catalytic domain of the hepatitis C virus NS2-3 protease. Nature. 2006;442(7104):831–5. Epub 2006/07/25. nature04975 [pii] doi: 10.1038/nature04975 . 16862121

[9] Santolini E, Pacini L, Fipaldini C, Migliaccio G, Monica N. The NS2 protein of hepatitis C virus is a transmembrane polypeptide. J Virol. 1995;69(12):7461–71. Epub 1995/12/01. doi: 10.1128/JVI.69.12.7461-7471.1995 . 7494252 PMC189684

[10] Jirasko V, Montserret R, Lee JY, Gouttenoire J, Moradpour D, Penin F, et al. Structural and functional studies of nonstructural protein 2 of the hepatitis C virus reveal its key role as organizer of virion assembly. PLoS pathogens. 2010;6(12):e1001233. doi: 10.1371/journal.ppat.1001233 ; PubMed Central PMCID: PMC3002993. 21187906 PMC3002993

[11] Boukadida C, Fritz M, Blumen B, Fogeron ML, Penin F, Martin A. NS2 proteases from hepatitis C virus and related hepaciviruses share composite active sites and previously unrecognized intrinsic proteolytic activities. PLoS pathogens. 2018;14(2):e1006863. doi: 10.1371/journal.ppat.1006863 ; PubMed Central PMCID: PMC5819835. 29415072 PMC5819835

[12] Schregel V, Jacobi S, Penin F, Tautz N. Hepatitis C virus NS2 is a protease stimulated by cofactor domains in NS3. Proceedings of the National Academy of Sciences of the United States of America. 2009;106(13):5342–7. Epub 2009/03/14. 0810950106 [pii] doi: 10.1073/pnas.0810950106 . 19282477 PMC2663979

[13] Isken O, Langerwisch U, Jirasko V, Rehders D, Redecke L, Ramanathan H, et al. A conserved NS3 surface patch orchestrates NS2 protease stimulation, NS5A hyperphosphorylation and HCV genome replication. PLoS pathogens. 2015;11(3):e1004736. doi: 10.1371/journal.ppat.1004736 ; PubMed Central PMCID: PMC4361677. 25774920 PMC4361677

[14] Boukadida C, Marnata C, Montserret R, Cohen L, Blumen B, Gouttenoire J, et al. NS2 proteins of GB virus B and hepatitis C virus share common protease activities and membrane topologies. J Virol. 2014;88(13):7426–44. doi: 10.1128/JVI.00656-14 ; PubMed Central PMCID: PMC4054453. 24741107 PMC4054453

[15] Lohmann V, Korner F, Koch J, Herian U, Theilmann L, Bartenschlager R. Replication of subgenomic hepatitis C virus RNAs in a hepatoma cell line. Science. 1999;285(5424):110–3. Epub 1999/07/03. 7638 [pii]. doi: 10.1126/science.285.5424.110 . 10390360

[16] Welbourn S, Green R, Gamache I, Dandache S, Lohmann V, Bartenschlager R, et al. Hepatitis C virus NS2/3 processing is required for NS3 stability and viral RNA replication. The Journal of biological chemistry. 2005;280(33):29604–11. Epub 2005/06/28. M505019200 [pii] doi: 10.1074/jbc.M505019200 . 15980068

[17] Wu MJ, Shanmugam S, Welsch C, Yi M. Palmitoylation of Hepatitis C Virus NS2 Regulates Its Subcellular Localization and NS2-NS3 Autocleavage. J Virol. 2019;94(1). doi: 10.1128/JVI.00906-19 ; PubMed Central PMCID: PMC6912101. 31597774 PMC6912101

[18] Popescu CI, Callens N, Trinel D, Roingeard P, Moradpour D, Descamps V, et al. NS2 Protein of Hepatitis C Virus Interacts with Structural and Non-Structural Proteins towards Virus Assembly. PLoS pathogens. 2011;7(2):e1001278. Epub 2011/02/25. doi: 10.1371/journal.ppat.1001278 . 21347350 PMC3037360

[19] Stapleford KA, Lindenbach BD. Hepatitis C virus NS2 coordinates virus particle assembly through physical interactions with the E1-E2 glycoprotein and NS3-NS4A enzyme complexes. J Virol. 2011;85(4):1706–17. doi: 10.1128/JVI.02268-10 ; PubMed Central PMCID: PMC3028914. 21147927 PMC3028914

[20] Ma Y, Anantpadma M, Timpe JM, Shanmugam S, Singh SM, Lemon SM, et al. Hepatitis C virus NS2 protein serves as a scaffold for virus assembly by interacting with both structural and nonstructural proteins. J Virol. 2011;85(1):86–97. doi: 10.1128/JVI.01070-10 ; PubMed Central PMCID: PMC3014171. 20962101 PMC3014171

[21] Phan T, Beran RKF, Peters C, Lorenz I, Lindenbach BD. Hepatitis C virus NS2 protein contributes to virus particle assembly via opposing epistatic interactions with the E1-E2 glycoprotein and NS3-4A enzyme complexes. J Virol. 2009:JVI.00891-09. doi: 10.1128/jvi.00891-09 19515772 PMC2738163

[22] Lorenz IC. The Hepatitis C Virus Nonstructural Protein 2 (NS2): An Up-and-Coming Antiviral Drug Target. Viruses. 2010;2(8):1635–46. doi: 10.3390/v2081635 ; PubMed Central PMCID: PMC3185728. 21994698 PMC3185728

[23] Yao N, Reichert P, Taremi SS, Prosise WW, Weber PC. Molecular views of viral polyprotein processing revealed by the crystal structure of the hepatitis C virus bifunctional protease-helicase. Structure. 1999;7(11):1353–63. doi: 10.1016/s0969-2126(00)80025-8 . 10574797

[24] Kim JL, Morgenstern KA, Lin C, Fox T, Dwyer MD, Landro JA, et al. Crystal structure of the hepatitis c virus NS3 protease domain complexed with a synthetic NS4A cofactor peptide. Cell. 1996;87:343–55. doi: 10.1016/s0092-8674(00)81351-3 8861917

[25] Dentzer TG, Lorenz IC, Evans MJ, Rice CM. Determinants of the hepatitis C virus nonstructural protein 2 protease domain required for production of infectious virus. J Virol. 2009;83(24):12702–13. doi: 10.1128/JVI.01184-09 ; PubMed Central PMCID: PMC2786863. 19812162 PMC2786863

[26] Reed KE, Grakoui A, Rice CM. Hepatitis C virus-encoded NS2-3 protease: cleavage-site mutagenesis and requirements for bimolecular cleavage. J Virol. 1995;69(7):4127–36. Epub 1995/07/01. doi: 10.1128/JVI.69.7.4127-4136.1995 . 7769671 PMC189148

[27] Pallaoro M, Lahm A, Biasiol G, Brunetti M, Nardella C, Orsatti L, et al. Characterization of the hepatitis C virus NS2/3 processing reaction by using a purified precursor protein. J Virol. 2001;75(20)):9939–46. doi: 10.1128/JVI.75.20.9939-9946.2001 11559826 PMC114565

[28] Foster TL, Belyaeva T, Stonehouse NJ, Pearson AR, Harris M. All three domains of the hepatitis C virus nonstructural NS5A protein contribute to RNA binding. J Virol. 2010;84(18):9267–77. Epub 2010/07/02. JVI.00616-10 [pii] doi: 10.1128/JVI.00616-10 . 20592076 PMC2937630

[29] Madan V, Paul D, Lohmann V, Bartenschlager R. Inhibition of HCV replication by cyclophilin antagonists is linked to replication fitness and occurs by inhibition of membranous web formation. Gastroenterology. 2014;146(5):1361–72 e1-9. doi: 10.1053/j.gastro.2014.01.055 . 24486951

[30] Foy E, Li K, Wang C, Sumpter R Jr., Ikeda M, Lemon SM, et al. Regulation of interferon regulatory factor-3 by the hepatitis C virus serine protease. Science. 2003;300(5622):1145–8. doi: 10.1126/science.1082604 . 12702807

[31] Koch JO, Bartenschlager R. Modulation of hepatitis C virus NS5A hyperphosphorylation by nonstructural proteins NS3, NS4A, and NS4B. J Virol. 1999;73(9):7138–46. Epub 1999/08/10. doi: 10.1128/JVI.73.9.7138-7146.1999 . 10438800 PMC104237

[32] Schultz DE, Honda M, Whetter LE, McKnight KL, Lemon SM. Mutations within the 5’ nontranslated RNA of cell culture-adapted hepatitis A virus which enhance cap-independent translation in cultured African green monkey kidney cells. J Virol. 1996;70(2):1041–9. doi: 10.1128/JVI.70.2.1041-1049.1996 ; PubMed Central PMCID: PMC189910. 8551562 PMC189910

[33] Takamizawa A, Mori C, Fuke I, Manabe S, Murakami S, Fujita J, et al. Structure and organization of the hepatitis C virus genome isolated from human carriers. J Virol. 1991;65(3)):1105–13. doi: 10.1128/JVI.65.3.1105-1113.1991 1847440 PMC239876

[34] Wakita T, Pietschmann T, Kato T, Date T, Miyamoto M, Zhao Z, et al. Production of infectious hepatitis C virus in tissue culture from a cloned viral genome. Nat Med. 2005;11(7):791–6. Epub 2005/06/14. nm1268 [pii] doi: 10.1038/nm1268 . 15951748 PMC2918402

[35] Isken O, Langerwisch U, Schonherr R, Lamp B, Schroder K, Duden R, et al. Functional characterization of bovine viral diarrhea virus nonstructural protein 5A by reverse genetic analysis and live cell imaging. J Virol. 2014;88(1):82–98. Epub 2013/10/18. doi: 10.1128/JVI.01957-13 ; PubMed Central PMCID: PMC3911689. 24131714 PMC3911689

[36] Sutter G, Ohlmann M, Erfle V. Non-replicating vaccinia vector efficiently expresses bacteriophage T7 RNA polymerase. FEBS letters. 1995;371:9–12. doi: 10.1016/0014-5793(95)00843-x 7664891

[37] Backes P, Quinkert D, Reiss S, Binder M, Zayas M, Rescher U, et al. Role of annexin A2 in the production of infectious hepatitis C virus particles. J Virol. 2010;84(11):5775–89. Epub 2010/03/26. JVI.02343-09 [pii] doi: 10.1128/JVI.02343-09 . 20335258 PMC2876593

[38] Powell MJD. Restart procedures for the conjugate gradient method. Mathematical Programming. 1977;12(1):241–54. doi: 10.1007/BF01593790

