# Supplementary figures and images for: Identification of NS2 determinants stimulating intrinsic HCV NS2 protease activity
Source: PLoS Pathog. 2022 Jun 21;18(6):e1010644. doi: 10.1371/journal.ppat.1010644 (PMC9249167; doi:10.1371/journal.ppat.1010644)

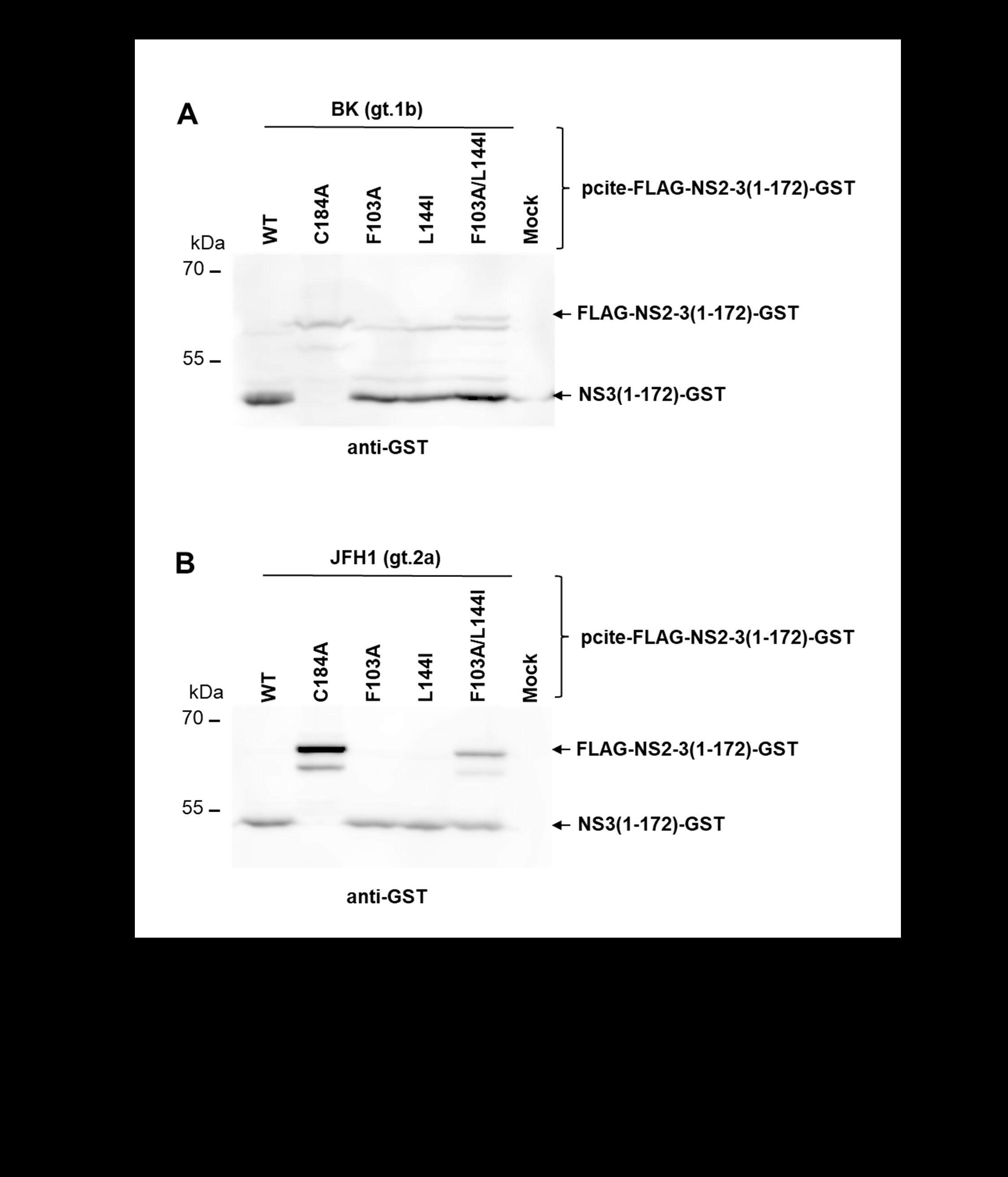

Supplement: S1 Fig — MVA/T7pol-infected Huh7-T7 cells were transfected with 4 μg of the indicated pcite-Flag-NS-NS3(1–172)-GST/BK (A) or pcite-Flag-NS-NS3(1–172)-GST/JFH1 (B) plasmid derivatives. Transfected cells were harvested 20 h post-transfection into lysate buffer. Afterwards, protein lysates were separated by SDS-PAGE and analyzed by Western blot using anti-GST antibody. Molecular mass standards are indicated on the left. Position of the uncleaved precursor Flag-NS2-NS3(1–172)-GST and cleavage product NS3(1–172)-GST are indicated by arrows on the right. WT: wild-type; mock: transfection control without DNA. (TIF) [file ppat.1010644.s001.tif]
